# The expanded inhibitor of apoptosis gene family in oysters possesses novel domain architectures and may play diverse roles in apoptosis following immune challenge

**DOI:** 10.1186/s12864-021-08233-6

**Published:** 2022-03-12

**Authors:** Erin M. Witkop, Dina A. Proestou, Marta Gomez-Chiarri

**Affiliations:** 1 Department of Fisheries, Animal and Veterinary Science, University of Rhode Island, Kingston, RI USA; 2 USDA ARS NEA NCWMAC Shellfish Genetics Program, Kingston, RI USA

**Keywords:** Transcriptome, Oyster, WGCNA, Inhibitor of apoptosis proteins (IAP), DESeq2, Gene expansion, Apoptosis, Immunity

## Abstract

**Background:**

Apoptosis plays important roles in a variety of functions, including immunity and response to environmental stress. The Inhibitor of Apoptosis (IAP) gene family of apoptosis regulators is expanded in molluscs, including eastern, *Crassostrea virginica*, and Pacific, *Crassostrea gigas,* oysters. The functional importance of IAP expansion in apoptosis and immunity in oysters remains unknown.

**Results:**

Phylogenetic analysis of IAP genes in 10 molluscs identified lineage specific gene expansion in bivalve species. Greater IAP gene family expansion was observed in *C. virginica* than *C. gigas* (69 vs. 40), resulting mainly from tandem duplications. Functional domain analysis of oyster IAP proteins revealed 3 novel Baculoviral IAP Repeat (BIR) domain types and 14 domain architecture types across gene clusters, 4 of which are not present in model organisms. Phylogenetic analysis of bivalve IAPs suggests a complex history of domain loss and gain. Most IAP genes in oysters (76% of *C. virginica* and 82% of *C. gigas*), representing all domain architecture types, were expressed in response to immune challenge (Ostreid Herpesvirus OsHV-1, bacterial probionts *Phaeobacter inhibens* and *Bacillus pumilus*, several *Vibrio* spp., pathogenic *Aliiroseovarius crassostreae*, and protozoan parasite *Perkinsus marinus*). Patterns of IAP and apoptosis-related differential gene expression differed between the two oyster species, where *C. virginica*, in general, differentially expressed a unique set of IAP genes in each challenge, while *C. gigas* differentially expressed an overlapping set of IAP genes across challenges. Apoptosis gene expression patterns clustered mainly by resistance/susceptibility of the oyster host to immune challenge. Weighted Gene Correlation Network Analysis (WGCNA) revealed unique combinations of transcripts for 1 to 12 IAP domain architecture types, including novel types, were significantly co-expressed in response to immune challenge with transcripts in apoptosis-related pathways.

**Conclusions:**

Unprecedented diversity characterized by novel BIR domains and protein domain architectures was observed in oyster IAPs. Complex patterns of gene expression of novel and conserved IAPs in response to a variety of ecologically-relevant immune challenges, combined with evidence of direct co-expression of IAP genes with apoptosis-related transcripts, suggests IAP expansion facilitates complex and nuanced regulation of apoptosis and other immune responses in oysters.

**Supplementary Information:**

The online version contains supplementary material available at 10.1186/s12864-021-08233-6.

## Introduction

Invertebrates lack the adaptive immune system of vertebrates and instead rely on complex innate immune systems with highly diverse (within and between species) gene families of pattern recognition receptors (PRRs) and effector molecules [1, 2]. Whole genome sequencing of several ecologically and economically important bivalve molluscs, including clams, mussels, oysters, and scallops, have revealed large-scale expansion and diversification of several immune gene families, including Toll-Like Receptors (TLRs), C1qDC proteins, Fibrinogen-related proteins (FREPs), and members of the Inhibitor of Apoptosis Proteins (IAP) family, also called BIR domain-containing (BIRC) proteins [3–10]. Transcriptomic studies in bivalves indicate expanded immune gene families display highly specific and orchestrated gene expression responses to biotic and abiotic stressors [3, 4, 6, 11–16]. These gene families may have undergone functional diversification, which is hypothesized to enhance the oyster’s ability to mount tailored immune responses to the variety of pathogens in their environment [3, 4, 17].

In oyster (Ostreida, Mollusca) species*,* apoptosis is critical for fighting viral, parasitic, and bacterial infections [18–20]. Apoptosis is a highly conserved form of regulated cell death mediated by two major pathways, the death-receptor mediated (extrinsic) pathway, and the mitochondrial (intrinsic) pathway [21]. Apoptosis pathways crosstalk extensively with other immune pathways, including inflammation mediated by Nuclear Factor-κB (NF-κB), autophagy, and alternative forms of cell death like necroptosis and parthanatos [21, 22]. In hemocytes, the major immune and phagocytic cell of the oyster, different immune stressors can stimulate or suppress apoptosis in unique ways, leading to varied pathological outcomes [20].

Inhibitor of Apoptosis proteins regulate cell death pathways by directly or indirectly inhibiting caspases, regulating ubiquitin (Ub)-dependent signaling events via E3 ligase activity, and mediating activation of the pro-survival NF-κB pathway [23, 24]. Mammals have 8 BIRC members; BIRC1 (NAIP), BIRC2 (cIAP1), BIRC3 (cIAP2), BIRC4 (XIAP), BIRC5 (Survivin), BIRC6 (BRUCE/Apollon), BIRC7 (ML-IAP), and BIRC8 (ILP2) [25], while *Drosophila melanogaster* contains two (DIAP1 and DIAP2) [26]. IAPs possess one to three N-terminal Baculovirus IAP Repeat (BIR) domains, which are classified as Type I or Type II [23]. The unique functions of IAPs are influenced by the number and combinations of Type I and Type II BIR repeats, and by the presence of key additional protein domains. Type II BIRs possess a hydrophobic deep peptide binding groove that binds caspases and IAP antagonists (i.e. Smac/DIABLO) that have N-terminal IAP binding motifs (IBMs). Type I BIRs interact instead with Tumor Necrosis Factor Receptor Associated Factor (TRAF) 1, TRAF2, and transforming growth factor-B activated kinase (TAK1) binding protein (TAB1), involved in promoting cell survival and NF-κB pathway activation [27–29]. IAPs can also possess Really Interesting New Gene (RING), ubiquitin-associated (UBA), ubiquitin-conjugating (UBC), and caspase activation and recruitment (CARD) domains. The RING, UBA, and UBC domains play critical roles in the ubiquitination cascade, where the UBC domain acts as an E2 ubiquitin-conjugating enzyme, the RING domain acts as an E3 ubiquitin ligase, and the UBA domain allows for binding of unique polyubiquitin chains. IAPs therefore also play critical roles in targeting proteins for proteasomal degradation and overall protein turnover [30].

Investigation of the IAP family in mammals has provided key insights into the unique and diverse roles of IAP members in cell death, immune regulation, and critical cellular processes such as cell migration and replication. BIRC4/XIAP inhibits apoptosis through direct physical binding with caspase 3, while BIRC2 and BIRC3 (cIAP1, cIAP2) do so through ubiquitination and promotion of proteasomal degradation [25]. BIRC2 and BIRC3 also mediate cell death or cell survival through signal transduction of death receptor binding (TNFR) during extrinsic apoptosis and canonical NF-κB pathway activation. BIRC2, BIRC3 and BIRC4 play roles in inflammasome regulation [24, 25, 31]. BIRC4, BIRC5, and BIRC6 have been shown to have a regulatory influence on autophagy [32]. BIRC7 and BIRC4 in mammals, as well as DIAP1 in *D. melanogaster,* can modulate cell migration [33, 34]. Finally, BIRC6 is involved in DNA double strand break repair, homologous recombination, and autophagosome-lysosome fusion independent of ubiquitination activity [35, 36]. Conservation of these functions in oysters and other bivalve molluscs, however, remains unknown.

Expansion of apoptosis pathway gene families, and the IAP family in particular, has been noted previously in molluscs [4, 6, 12, 37]. Transcriptome studies in the Pacific oyster, *Crassostrea gigas* (Ostreida)*,* and the eastern oyster, *C. virginica* (Ostreida)*,* indicate IAP family members significantly respond to viral challenge with Ostreid Herpesvirus type 1 (OsHV-1, which causes mortality in Pacific oysters), bacterial challenge with *Aliiroseovarius crassostreae* (causative agent of Roseovarius or Juvenile Oyster Disease, ROD/JOD, in eastern oysters) and *Vibrio* spp. (causative agent of larval vibriosis in bivalves), and parasitic challenge with the parasite *Perkinsus marinus* (causative agent of Dermo disease in eastern oysters) [12, 16, 38–42]. However, the role of IAP gene expansion in oyster immune responses remains unknown. Comparison of the usage of this expanded family across a diverse set of immune challenges from economically and ecologically relevant pathogens may provide insights into the role of IAP gene expansion in oysters’ ability to tailor and diversify their immune responses to unique challenges [11]. This study therefore assesses IAP genetic diversity across 10 sequenced mollusc genomes that span the phylogeny of Mollusca, explores potential mechanisms contributing to gene family expansion, and characterizes IAP domain architecture diversity in two oyster species. Furthermore, patterns of IAP differential expression were investigated in eight publicly available oyster immune challenge transcriptome datasets and correlations between IAP and apoptosis pathway expression were identified by Weighted Gene Correlation Network Analysis (WGCNA). This research sheds light on the potential role of IAP family diversification in apoptotic and immune responses, improves our understanding of how gene family expansion contributes to diverse immune responses in invertebrates, and informs future development of IAP candidate markers associated with apoptosis and disease resistance.

## Results

### Patterns of IAP gene family expansion in molluscs

**Fig. 1:**
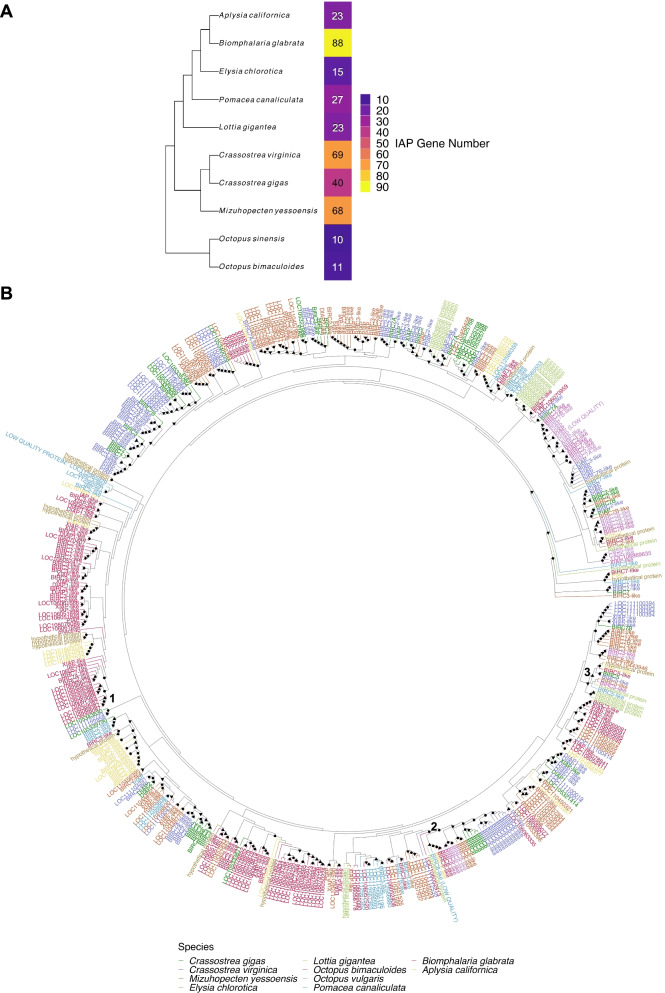
IAP expansion across Mollusca shows complex species-specific expansion and cross-species conservation. IAP proteins were identified using HMMER and Interproscan across 10 molluscan annotated genomes (*Aplysia californica, Biomphalaria glabrata,* and *Elysia chlorotica* (Heterobranchia), *Crassostrea virginica* and *Crassostrea gigas* (Ostreida), *Lottia gigantea* (Patellogastropoda), *Mizuhopecten yessoensis* (Pectinida), *Octopus bimaculoides* and *Octopus vulgaris* (*sinensis*) (Octopoda), and *Pomacea canaliculata* (Coengastropoda), Table 1) and patterns of IAP protein expansion were assessed by generating phylogenetic trees. **A** Phylogenetic tree of studied mollusc genomes produced by OrthoFinder with a heatmap depicting the number of IAP genes in each species. IAPs were most expanded in *B. glabrata*, least expanded in *Octopus* spp., and more expanded in *C. virginica* than *C. gigas*. **B** Phylogenetic tree of the longest isoform IAP transcript sequences across 10 mollusc species produced with RAxML and aligned with MAFFT. Sequences are named with shortened RefSeq product names or gene locus identifiers for those annotated as “uncharacterized protein LOCX”. Node shapes indicate bootstrap support (circle = 90–100, upward triangle = 70–89, downward triangle = 50–69) and numbers indicate clusters of interest referred to in text. IAP proteins clustered mainly by species-relationship but presented species-specific clusters, with *B. glabrata* having the largest (cluster 1). BIRC6-like (cluster 2) and BIRC5-like (cluster 3) proteins from all studied molluscan species clustered closely together, suggesting potential cross-species conservation

To better understand the degree of IAP expansion in oysters, IAP proteins were identified via the presence of BIR domains in protein sequences across 10 molluscan genomes using HMMER and Interproscan analysis. These representative genomes span the phylogeny of Mollusca: *Aplysia californica* (Heterobranchia), *Biomphalaria glabrata* (Heterobranchia), *Crassostrea virginica* (Ostreida), *Crassostrea gigas* (Ostreida), *Elysia chlorotica* (Heterobranchia), *Lottia gigantea* (Patellogastropoda), *Mizuhopecten yessoensis* (Pectinida), *Octopus bimaculoides* (Octopoda), *Octopus vulgaris* (*sinensis*) (Octopoda), *Pomacea canaliculata* (Coengastropoda) (Table 1). Following HMMER analysis and pruning of proteins lacking BIR domains as identified by Interproscan, 791 IAP transcripts were identified across all studied mollusc annotated genomes. The *C. virginica* reference genome (V 3.0, GCA_002022765.4) contained 69 genes and 158 IAP transcripts while the *C. gigas* reference genome (V 9.0, GCA_000297895.1) contained 40 genes and 74 IAP transcripts. Pruning this transcript list to remove isoforms with the same amino acid sequence yielded 84 *C. virginica* IAP transcripts and 45 *C. gigas* transcripts. The gastropod *B. glabrata* showed the greatest IAP gene expansion, with 88 genes, while cephalopods *O. vulgaris* (*sinensis*) and *O. bimaculoides* showed the fewest genes, with 10 and 11, respectively (Fig. 1a).

**Table 1:** Molluscan genome metadata

Organism Name	Class	Subclass/Order	Common Name	Phylum	Assembly	Version	Level	Size (Mb)	GC%	WGS	Scaffolds	CDS	Release Date
*Crassostrea gigas*	Bivalvia	Ostreida	Pacific oyster	Mollusca	GCA_000297895.1	9	Scaffold	557.736	35.3	AFTI01	7659	46,753	2012–09-17 T00:00:00Z
*Crassostrea virginica*	Bivalvia	Ostreida	eastern oyster	Mollusca	GCA_002022765.4	3	Chromosome	684.741	34.8191	MWPT03	11	60,213	2017–03-07 T00:00:00Z
*Mizuhopecten yessoensis*	Bivalvia	Pectinida	japanese scallop	Mollusca	GCA_002113885.1	2	Scaffold	987.589	37.6001	NEDP02	82,659	41,567	2017–04-27 T00:00:00Z
*Octopus bimaculoides*	Cephalopoda	Octopoda	california two spot octopus	Mollusca	GCA_001194135.1	2	Scaffold	2338.19	37.8	LGKD01	151,674	23,994	2015–07-31 T00:00:00Z
*Octopus vulgaris (sinensis)*	Cephalopoda	Octopoda	east asian common octopus	Mollusca	GCA_006345805.1	1	Chromosome	2719.15	36.3702	VCDQ01	13,516	25,656	2019–06-14 T00:00:00Z
*Aplysia californica*	Gastropoda	Heterobranchia	california sea hare (sea slug)	Mollusca	GCA_000002075.2	3	Scaffold	927.31	41.9999	AASC03	4332	27,608	2006–08-17 T00:00:00Z
*Biomphalaria glabrata*	Gastropoda	Heterobranchia	marsh snail	Mollusca	GCA_000457365.1	1	Scaffold	916.388	36.1998	APKA01	331,401	36,675	2013–08-09 T00:00:00Z
*Elysia chlorotica*	Gastropoda	Heterobranchia	eastern emerald elysia	Mollusca	GCA_003991915.1	2	Scaffold	557.48	36.5	RQTK01	9989	23,871	2019–01-04 T00:00:00Z
*Lottia gigantea*	Gastropoda	Patellogastropoda	owl limpet	Mollusca	GCA_000327385.1	1	Scaffold	359.506	36	AMQO01	4469	23,822	2012–12-20 T00:00:00Z
*Pomacea canaliculata*	Gastropoda	Caenogastropoda	golden apple snail	Mollusca	GCA_003073045.1	1	Chromosome	440.16	40.6223	PZQS01	24	40,391	2018–04-26 T00:00:00Z

Metadata regarding the mollusc species and genome versions utilized to study patterns of IAP expansion across molluscs. Class and Subclass/Order, assembly accession, genome version, and assembly statistics were provided by NCBI

A phylogenetic tree of IAP amino acid sequences revealed a complex pattern of species-specific expansions and cross-species conservation of IAP proteins (Fig. 1b). In general, this phylogeny recapitulated evolutionary relationships in molluscs, with *Octopus* spp. as the sister group, separation between bivalve (*C. gigas*, *C. virginica*, and *M. yessoensis*) and non-bivalve molluscs (*B. glabrata*, *E. chlorotica*, *A. californica*, *P. canaliculata*), and IAPs from sister species mostly clustered together (Fig. 1a) [43]. Each species had at least one well-supported (> 70 bootstrap support) species-specific protein cluster, and *B. glabrata* had the largest (cluster 1, Fig. 1b). Many well supported nodes (41 total) contained proteins from multiple species, including two conserved protein clusters (clusters 2 and 3) containing sequences from all but one molluscan species. The first multispecies cluster (cluster 2) contains proteins annotated as BIRC6 (or “hypothetical protein” in *L. gigantea*) from all species except *E. chlorotica*. The second conserved cluster (cluster 3) contains proteins annotated as BIRC5 (or “hypothetical protein” in *E. chlorotica* and *L. gigantea*) in all species except *O. bimaculoides*. Clustering of BIRC6 and BIRC5 proteins across molluscan species suggests sequence (and potentially functional) conservation in these two proteins.

### Potential bivalve IAP gene family expansion by tandem duplication and retroposition

The genomic distribution of *C. virginica* IAP genes across its 10 chromosomes and the presence of domains involved in retroposition in IAP genes across molluscs were investigated to assess potential mechanisms of IAP gene family expansion. *C. virginica* IAP genes were distributed across 9 of the 10 chromosomes, with the majority located on chromosomes 6 and 7 (Supplementary Fig. 1). IAP genes on chromosomes 6 and 7 were present in tandem arrays, suggesting tandem duplication as a mechanism of expansion, while genes present on other chromosomes were typically single genes.

Retroposition has been previously described as a mechanism of gene duplication in molluscs, with gene duplicates resulting from retroposition showing a lack of introns and a random distribution across genomes [6, 44, 45]. *L. gigantea* had the largest number of intronless genes [12], *C. virginica* had the second most [8], *C. gigas* had 3, *B. glabrata* had 2, and *M. yessoensis*, *O. vulgaris*, and *E. chlorotica* had one each (not shown). The 8 intronless *C. virginica* IAP genes were located on chromosomes 5, 7, 8, and 10 (Supplementary Fig. 1).

**Fig. 2:**
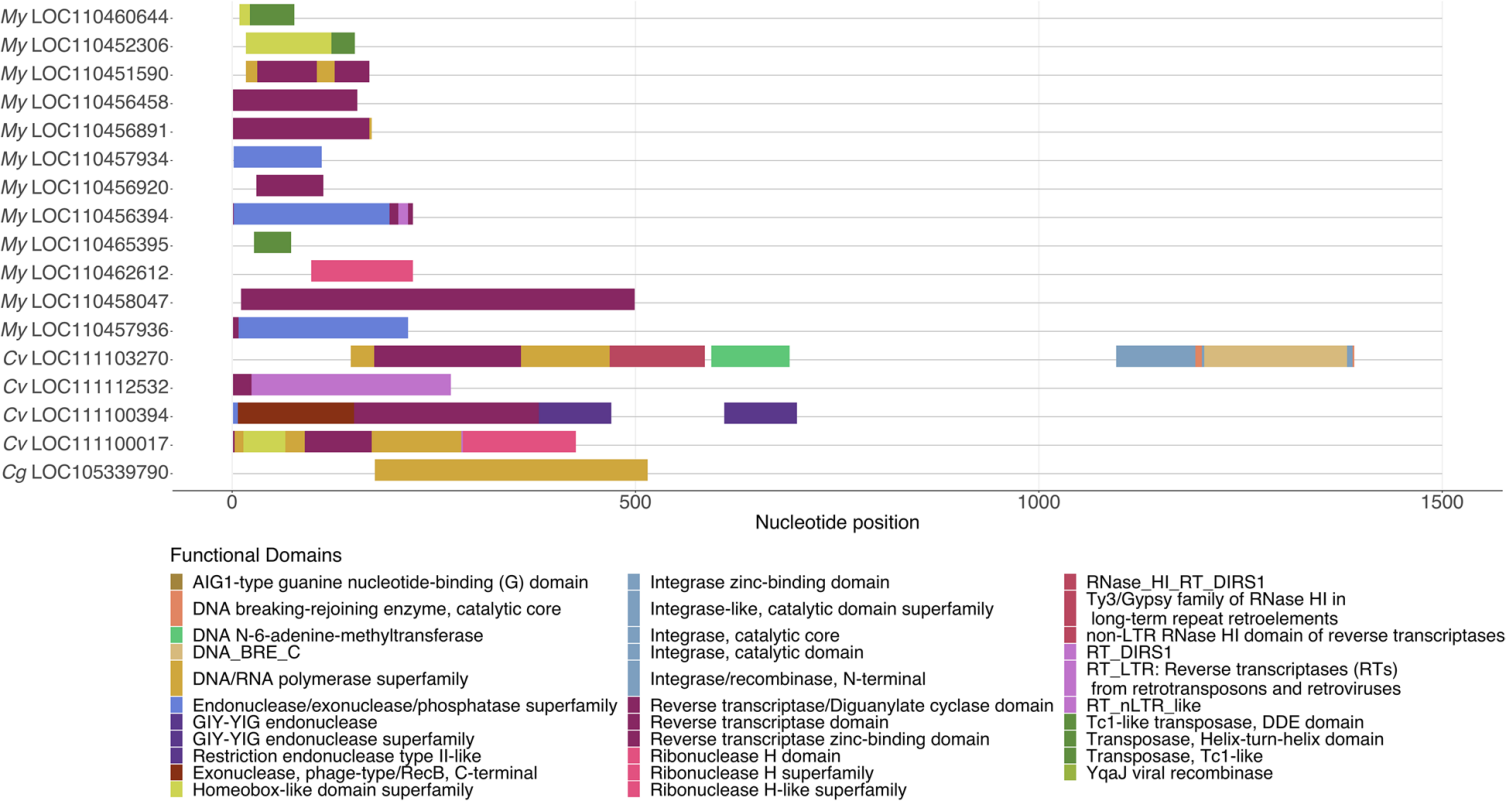
Retroposition may have been involved in IAP gene family expansion in oysters and the Chinese scallop. Functional domains involved in retrotransposition were identified in the translated open reading frames of oysters *Crassostrea gigas* (Cg)*, C. virginica* (Cv), and the Chinese scallop *Mizuhopecten yessoensis* (My) IAP genes using Interproscan. Functional domains are distinguished by color and plotted according to their position within the gene. Several oyster and scallop IAP genes possess functional domains necessary for active retrotransposition. This evidence, coupled with presence of intronless IAP genes, suggests retroposition as a potential mechanism of IAP expansion in molluscan bivalves

The presence of domains suggesting functional retroposition and transposition machinery in IAP bivalve gene sequences was investigated in the two oyster species, *C. virginica*, *C. gigas*, using as an outgroup the most closely related bivalve outside oysters within the 10 representative molluscan genomes, the scallop *M. yessoensis* (Pectinida) (Fig. 2). Functional domain analysis of translated IAP gene open reading frames (ORFs) revealed four *C. virginica* IAP genes contained domains involved in LTR and non-LTR retroposition, none of them intronless. *M. yessoensis* ORFs across nine genes also contained retroposition machinery and three possessed DNA transposase machinery (Transposase Tc-1 like domains: IPR002492, IPR027805, IPR038717, LOC110460644, LOC110452306, LOC110465395). The *C. gigas* genome assembly (V9.0, GCA_000297895.1) only contained one IAP gene with potential retroposition machinery, a reverse transcriptase domain (Fig. 2). This result suggests that retroposition may be common in bivalves and may be responsible for some of the IAP diversity observed in these species.

### Oyster IAPs contain conserved and novel BIR domain types

**Fig. 3:**
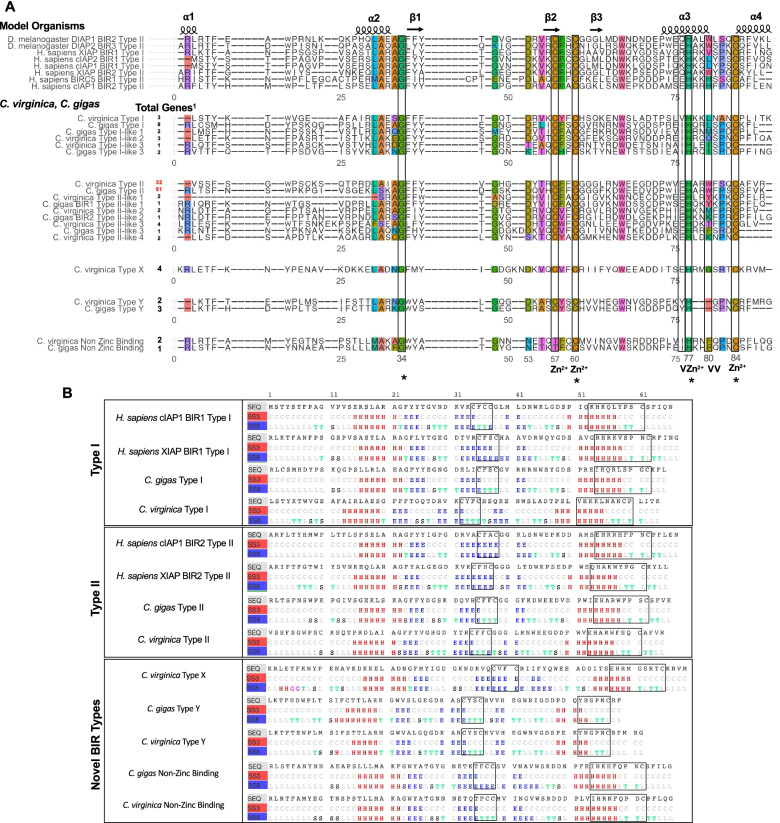
Oyster IAPs possess conserved and novel BIR domains with potentially altered secondary structure. **A** Alignment (MAFFT) of representative Type I and Type II BIR-defining amino acid sequences (highlighted with black boxes) of selected vertebrate and invertebrate model organisms (*Drosophila melanogaster*, *Homo sapiens*, *Mus musculus, Danio rerio*) with oyster IAP sequences revealed oysters possess both conserved and novel Type I and Type II domains. Three novel types of BIR domains (Type X, Type Y, and Type NZBIR) were identified in oysters. “Total Genes” indicate the number of oyster IAP genes with each identified BIR domain type, with the most represented highlighted in red. * = Conserved aa positions across all *C. gigas* (Ostreida) and *C. virginica* (Ostreida) BIR sequences. Zn^2+^ = positions in model organisms involved in Zinc atom stabilization. V = variable aa position used in BIR domain classification. **B** Predicted protein secondary structure analysis by RaptorX. Secondary structure predictions were made at the three class (SS3, red bar, H = alpha helix, E = beta sheet, C = coil) and eight class levels (SS8, blue bar, H = alpha helix, G = five-turn helix, I = extended strand in beta ladder, E = isolated beta bridge, T = hydrogen bonded turn, S = bend, L = loop) for representative BIR type amino acid sequence examples (SEQ, grey bar). Characteristic regions used in classification are outlined in black. Type X and Type BIRs may have shortened alpha helix structures while Type NZBIR does not have altered secondary structure but loss of Cysteine may prevent Zinc coordination

To assess the potential functional diversity of oyster IAPs, oyster BIR sequences were compared to the IAP-defining BIR Type I and Type II BIR domains from the best studied reference model organisms to include a range of BIR domain diversity across taxa in vertebrates (*Homo sapiens*, *Mus musculus, Danio rerio*), and one invertebrate *D. melanogaster* [46]. In *D. melanogaster* and *H. sapiens*, BIR domains are characterized by 15 conserved amino acids forming a central 3-stranded antiparallel β-sheet (β1–3) surrounded by 5 α-helices (α1–5), with four critical residues stabilizing a central zinc atom: Histidine (H77) and three Cysteine residues (C57, C60, C84) [46–48] (Fig. 3a). Multiple sequence alignment of all *C. virginica* and *C. gigas* BIR domain sequences identified by the Conserved Domain Database (CDD) via Interproscan and representative Type I and Type II sequences from the model organisms above revealed that only 4 (G34, C60, H77, C84) of the 15 conserved positions considered essential for BIR function in model organisms [46] were shared across all *C. gigas* and *C. virginica* proteins (Fig. 3, Supplementary Fig. 2). This result underscores extensive BIR domain diversity in oysters. Using amino acids in the α-3 and α-4 helix regions, oyster BIR sequences were classified as conserved Type I (H77, V80 or L80, C84) and conserved Type II (E76 or Q76, H77, W80 or H80, C84) [46, 47, 49] (Fig. 3, Supplementary Fig. 2). BIR repeats were additionally classified as Type I-like, with four Type-I like polymorphisms, if they had a hydrophobic residue at position 80 (I, V or L) and/or a Serine in position 81. Oyster sequences with an E76 prior to the conserved H77 were classified as Type II-like BIR repeats consistent with the arrangement observed in model organism Type II α-3 helices. In both *C. virginica* and *C. gigas*, conserved Type II repeats were the most common (Fig. 3a).

BIR sequences containing unique amino acids at key positions were classified as novel types (Fig. 3a,b, Supplementary Fig. 2). Two potentially functional (i.e. Zn-binding) novel BIR domain types were identified. Sequences with Glycine and Arginine substitutions at positions 80 and 82 respectively were observed in four *C. virginica* IAP genes and called Type X BIR. Alteration of secondary structure is not predicted for the Arginine substitution; however, shortening of the α-3 helix is predicted for the Glycine substitution (Fig. 3b). Type Y BIR, identified in two *C. virginica* and three *C. gigas* IAP genes, also are predicted to have a shortened alpha-helix secondary structure due to the loss of three amino acids, including conserved position 80 (Fig. 3b).

A final, but potentially non-functional, novel BIR type in *C. gigas* and *C. virginica* was identified by hydrophilic Threonine amino acid substitution at the first coordinating Cysteine residue (C57) of this zinc-binding structural hot spot [46–48]. Though this substitution is not predicted to alter protein secondary structure, loss of this Cysteine may result in decreased ability for these domains to coordinate with Zinc [46–48]; therefore, this domain is referred to as Non-Zinc Binding (NZBIR) here (Fig. 3b). IAP genes containing novel BIR types were rare in *C. virginica* and *C. gigas* (from 1 to 4 Fig. 3a), were distributed across the phylogenetic tree of IAP gene sequences, and did not group by type, suggesting they may have arisen independently across multiple IAPs (Supplementary Fig. 3a).

The number of BIR domains in each protein was also assessed to determine potential patterns of domain loss or gain over time. Most *C. virginica* IAP genes with CDD-identified BIR domains contained one BIR domain, while most *C. gigas* genes contained two (Supplementary Fig. 3b). Comparison of domain number across a phylogenetic tree of IAP nucleotide gene sequences suggests a pattern of BIR domain loss over time in *C. virginica* compared to *C. gigas* and *M. yessoensis* (Supplementary Fig. 3a).

### Oyster IAPs also present novel diversity in domain architectures

**Fig. 4:**
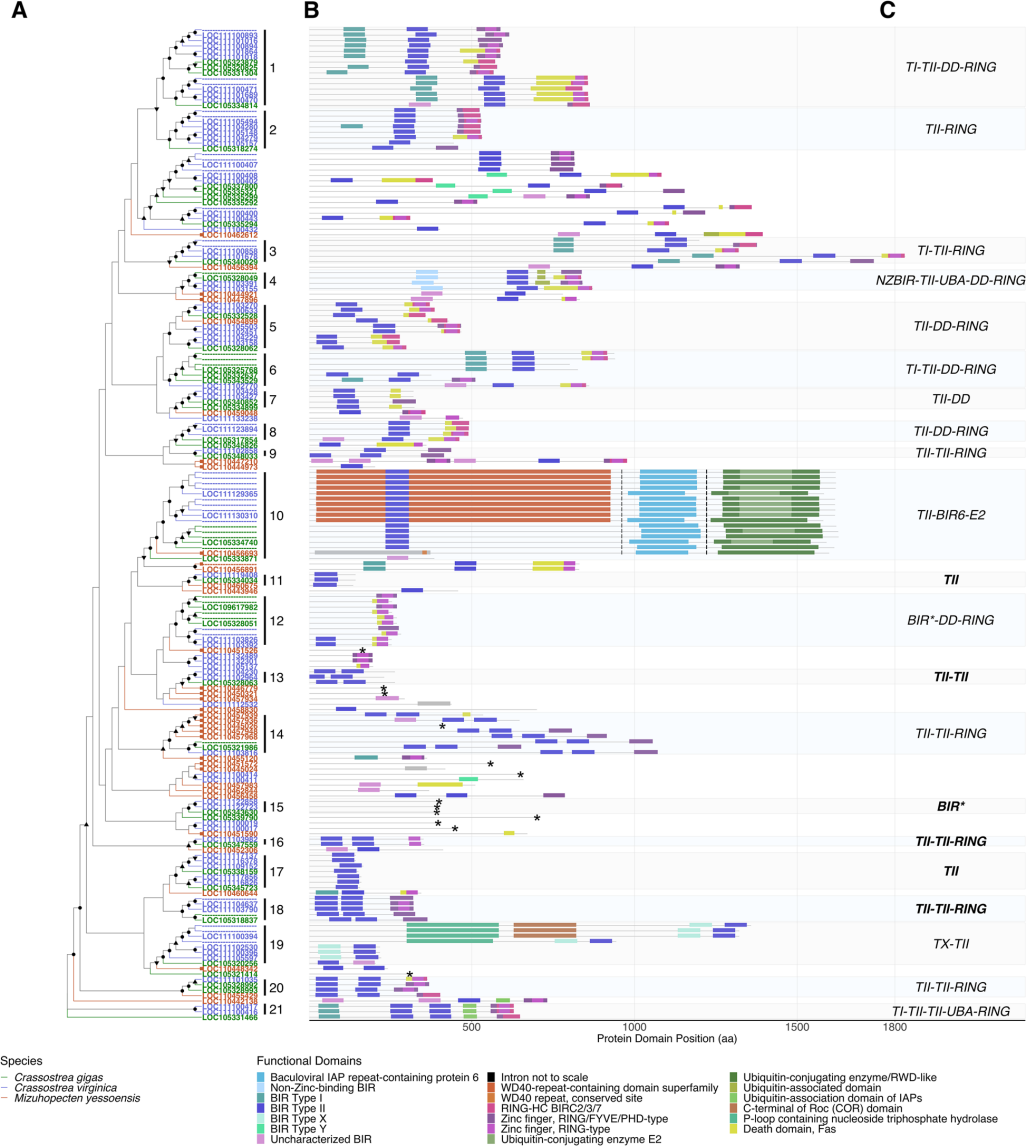
Diverse and novel protein domain architectures in the expanded scallop and oyster IAP gene family. **A** Phylogenetic tree of IAP amino acid sequences labelled by their gene ID in *C. gigas* (Ostreida) (green), *C. virginica* (Ostreida) (blue), and *M. yessoensis* (Pectinida) (orange). A square node tip indicates collapsed *M. yessoensis* sequences for improved visualization. Node shapes indicate bootstrap support (circle = 90–100, upward triangle = 70–89, downward triangle = 50–69). When multiple transcripts from the same gene clustered together, all but one were labelled with a “---”. “---”. IAPs grouped into 21 well supported clusters. **B** Functional domain architecture of each transcript isoform plotted by amino acid position with domains labeled by color. Asterisk indicates transcripts where IAP repeats were only identified by Interproscan and not CDD. Shaded boxes surround each well supported cluster. **C**. Domain architecture type for each cluster (TI = Type I BIR, TII = Type II BIR, UBA = UBA domain, RING = RING domain, DD = Death domain, BIR* = BIR domain identified by Interproscan and not CDD). Clusters where architecture was conserved between all proteins were labelled in bold. Clusters were classified into 14 domain architecture types, 4 of which are not found in the model organisms *D. melanogaster* or *H. sapiens*

In addition to variation in BIR domain primary structure, distinct domain architecture types shared across well-conserved protein phylogenetic clusters were examined to further characterize the potential functional breadth of expanded oyster IAPs. Interproscan analysis of oyster IAP amino acid sequences identified 12 non-BIR functional domains (Fig. 4). Many IAPs contained carboxyl terminus RING-finger domains (cd16713, RING-HC_BIRC2_3_7; IPR013083, Zinc finger, RING/FYVE/PHD-type; IPR001841, Zinc finger, RING-type) and death domain (DD) architecture (G3DSA:1.10.533.10, Death Domain, Fas). Several proteins in *C. virginica* and *C. gigas* contained UBA (IPR015940, Ubiquitin-associated domain; cd14321 UBA domain found in inhibitor of apoptosis proteins (IAPs)), or UBC (IPR016135, Ubiquitin-conjugating enzyme/RWD-like; IPR000608, Ubiquitin-conjugating enzyme E2) domains. BIRC6-like proteins contained the characteristic BIRC6 domain (IPR022103, Baculoviral IAP repeat-containing protein 6) and a UBC domain (IPR000608), but only contained WD-40 repeat domains (IPR019775, WD40 repeat, conserved site; IPR036322, WD40-repeat-containing domain superfamily) in *C. virginica*. No CARD domains, a subfamily of DD characteristic of model species IAPs, were identified by Interproscan in any studied mollusc IAP [50].

**Fig. 5:**
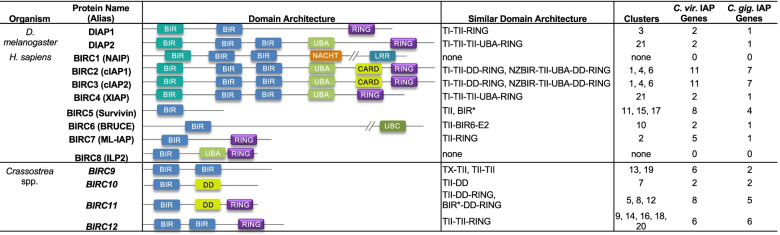
Diversity of domain structure in oyster IAPs as compared to IAPs in representative model organisms. *C. virginica* (*Cv*) and *C. gigas* (*Cg*) showed a diversity of IAP domain architectures, including several novel types. Top panel: Comparison of IAP domain architecture types conserved between oysters (column 4) and two model organisms with well characterized IAPs, *D. melanogaster* and *H. sapiens* [26, 51] (column 3). Column 5: ID of the clusters from Fig. 4 showing that architecture. Columns 6 and 7: number of oyster genes showing that architecture. Bottom panel: Novel domain architectures (named BIRC9, BIRC10, BIRC11, and BIRC12) only found in oysters

Analysis of the arrangement of conserved protein domains within oyster and scallop IAP amino acid sequences identified 14 distinct domain architecture types distributed across 21 well-supported phylogenetic protein clusters (> 90 bootstrap support) (Fig. 4a). Domain architecture types were defined by the order and number of functional domains identified by Interproscan (Fig. 4b,c). Ten of the 21 clusters contained proteins with domain architectures similar to those observed in humans or *D. melanogaster* (referred to as BIRC#-like). Novel architectures were identified in the remaining 11 clusters, named here BIRC9, BIRC10, BIRC11, and BIRC12 (Fig. 5). The BIRC2/3-like (defined here as 2 BIR domains, a DD, and a RING domain, or 2 BIR domains, a DD, a UBA, and a RING domain, assuming a similar function of DD architecture to the CARD domain [50]) and BIRC6-like domain architectures were most common across IAP genes in both oyster species, followed by BIRC11 in *C. virginica* and BIRC12 in *C. gigas* (Supplementary Table 1).

The four oyster IAPs containing a novel NZBIR domain were in cluster 4 (Fig. 4a). Three of these also contained a UBA, DD, and RING domain, most resembling the domain architecture of BIRC2/3 in mammals (though missing one TII domain). Therefore, oyster BIRC2/3-like showed two alternative domain structures: one containing TI-TII-DD-RING domains (clusters 1 and 6), and another that also contains a UBA domain, but in which the TI BIR domain seen in mammals is replaced by NZBIR (cluster 4; Fig. 4). *C. virginica* Type X sequences were located in cluster 19 (Fig. 4a). Genes containing the novel Type Y BIR domain were not present in a well-supported cluster and were not named. Three sequences with Type Y BIR domains also possessed a Type II BIR domain, all possessed a RING domain, and one possessed a RING and DD. Intronless *C. virginica* and *C. gigas* IAP genes (suspected to have arisen from retroposition) were located in protein clusters 17 and 13 and were all BIRC5-like with a single BIR domain (Fig. 4a).

Transcript evaluation indicated that alternative splicing provided an additional source of diversity in domain architectures, with some alternatively spliced transcripts from the same gene having varied functional domains (e.g.*,* cluster 3 LOC111100858, cluster 4 LOC105328049, Fig. 4). Comparison of domain architecture diversity across oysters suggests a complex history of domain loss and gain, and the large diversity of IAP domain architectures observed indicates the potential for varied functionality across oyster IAPs that surpasses that present in selected model organisms (*H. sapiens*, and *D. melanogaster*) with well characterized IAPs [26, 51].

### Almost the full spectrum of diversity in IAP domain architecture types characterized in oysters was expressed in response to immune challenge

**Fig. 6:**
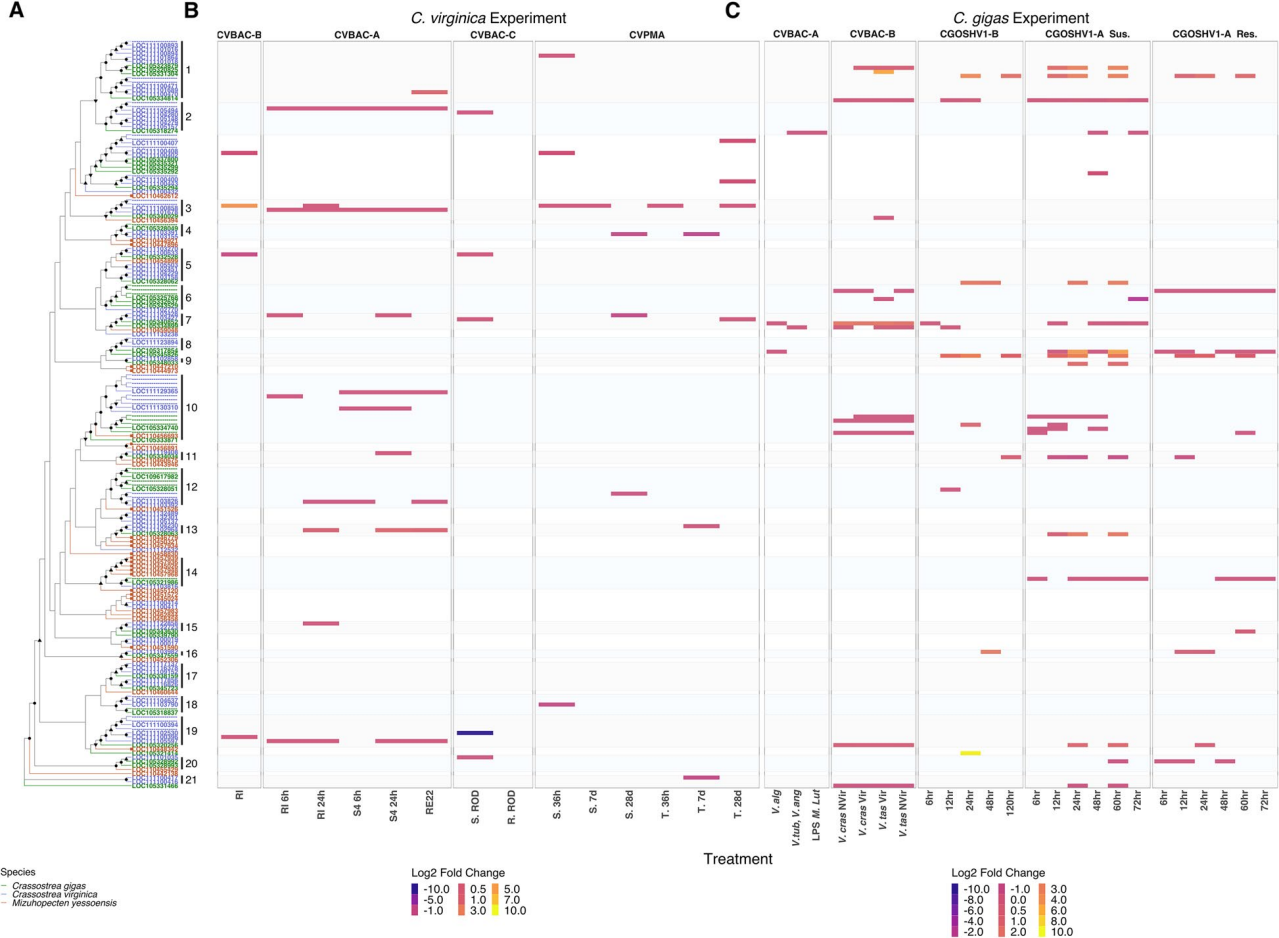
Complex patterns of IAP domain architecture and gene expression across immune challenges in oysters*.* Comparison of IAP differential gene expression patterns across 8 transcriptomes of *C. gigas* and *C. virginica* immune challenges revealed complex expression patterns of unique and shared gene sets across experiments. *C. virginica* expressed more unique genes and transcripts in each experiment than *C. gigas* All domain architectures, including novel architectures, were significantly differentially expressed in at least one oyster, and experiments expressed unique assemblages of multiple IAP domain architectures. **A** Phylogenetic tree of IAP amino acid sequences labelled by their gene name in *C. gigas* (Ostreida) (green), *C. virginica* (Ostreida) (blue), and *M. yessoensis* (Pectinida) (orange). A square node tip indicates collapsed *M. yessoensis* proteins for the purpose of plotting. Node shapes indicate bootstrap support (circle = 90–100, upward triangle = 70–89, downward triangle = 50–69). Vertical bars indicate well-supported protein clusters (from Fig. 4a). Transcripts with the same amino acid sequence were collapsed by RAxML when producing the tree. Multiple transcript sequences from the same gene are named once on the lowest node and then represented by dashes (“----”). **B C** Heatmap of log2 fold change expression of significantly differentially expressed *C. virginica* (**B**) and *C. gigas* (**C**) IAPs in response to various immune challenge experiments (columns) plotted for each corresponding transcript in the phylogenetic tree. BAC: bacterial challenge. PM*:* parasitic challenge (*Perkinsus marinus*). OSHV1: viral challenge. Sus: susceptible oysters. Res: resistant oysters. Shaded boxes surround well supported protein clusters from (**A**)

Potential roles for oyster IAP gene family expansion, variation in BIR domain primary structure, and domain architecture diversity in innate immunity were investigated by comparing patterns of IAP differential expression in response to distinct immune challenges using 8 publicly available transcriptome datasets (NCBI SRA) (Table 2). Transcriptome sequencing revealed that most of the oyster IAP diversity is expressed in response to immune challenge, both in terms of domain architecture and overall IAP gene usage. However, expression patterns differed by oyster species and challenge type, suggesting diversity may have functional relevance in allowing responses to different conditions. Across the four *C. virginica* immune challenge experiments, 53 (77%) of the 69 IAP genes were expressed; 15 significantly differentially expressed compared to non-challenged controls (Fig. 6), 28 constitutively expressed (i.e. not significantly different to controls but expressed in every sample; Supplementary Fig. 4), and 10 genes with a mix of differential and constitutive gene expression. In contrast, in the four *C. gigas* immune challenge experiments, 33 (82%) of the 40 genes were expressed, with 20 differentially expressed, 8 constitutively expressed, and 5 genes with a mix of transcripts differentially or constitutively expressed (Fig. 6, Supplementary Fig. 4).

**Table 2:** Immune challenge transcriptome experiments

Experiment	Host Life Stage	Challenge Method	Host Stock	Challenge Type	Challenge Species	Sample Collection Post-Challenge	Citation
**CVBAC-A**	Veliger larvae	Laboratory	Mixed stock	Bacterial	Probionts *Phaeobacter inhibens* S4 and *Bacillus pumilus* RI06–95, or pathogenic *Vibrio coralliilyticus* RE22	6 h (RE22, RI, S4) or 24 h (S4, RI)	Modak et al., 2020 [52]
**CVBAC-B**	Veliger larvae	Hatchery	Mixed stock	Bacterial	Probionts *Phaeobacter inhibens* S4 and *Bacillus pumilus* RI06–95	5, 12, 16 d	Modak et al., 2020 [52]
**CVBAC-C Res., CVBAC-C Sus.**	Juvenile	Laboratory	Comparison of susceptible and resistant families	Bacterial	*Aliiroseovarius crassostreae*	1,5,15, 30 d	McDowell et al., 2014 [38]
**CVPMA Tol., CVPMA Sus.**	Adult	Laboratory	Comparison of susceptible and tolerant families	Parasitic	*Perkinsus marinus*	36 h, 7 d, 28 d	Proestou and Sullivan, 2020 [53]
**CGBAC-A**	Adult	Laboratory	Mixed stock	Bacterial	*V. anguillarum*, *V. tubiashii*, *V. aestuarianus*, *V. alginolyticus*-1, *V. alginolyticus*-2, *Micrococcus luteus*, LPS	12 h	Zhang et al. 2015 [3]
**CGBAC-B**	Juvenile	Laboratory	Mixed stock	Bacterial	Pathogenic and non-pathogenic *V. tasmaniensis* (LGP32) and *V. crassostreae* (J2–9)	8 h	Rubio et al. 2019 [54]
**CGOSHV1-A Res., CGOSHV1-A Sus.**	Juvenile	Field	Comparison of susceptible and resistant families	Viral	OsHV-1	0, 6, 12, 24, 48, 60, 72 h	de Lorgeril et al., 2018 [55]
**CGOSHV1-B**	Juvenile	Laboratory	Susceptible stock	Viral	OsHV-1	0, 6, 12, 24, 48, 120 h	He et al., 2015 [15]

Oyster transcriptome datasets were selected from publicly available data (NCBI SRA) from bacterial, parasitic, and viral challenges from a range of life stages (larval, juvenile, adult) and challenge methods (experimental and natural). Experiments were coded by their species (CV = *C. virginica*, CG = *C. gigas*), experimental challenge (BAC = bacterial, PMA = *Perkinsus marinus*, OSHV-1 = OsHV-1) and a letter code when there were multiple experiments of that type (A, B, C)

Differential gene expression of IAPs was seen in all oyster immune challenge experiments, but widely ranged in the number of differentially expressed IAP transcripts per experiment between 5 (CVBAC-B) and 32 (CVBAC-A) in *C. virginica* and 5 (CGBAC-A) and 68 (CGOSHV1-A Susceptible) in *C. gigas* (Supplementary Table 2). Greater gene expression overlap was seen across experiments in *C. gigas* than *C. virginica*, and 87% of differentially expressed genes were shared between *C. gigas* challenge experiments, compared to 48% in *C. virginica*. *C. gigas* also expressed more of the same transcripts across challenges than *C. virginica*, with 67% (CGBAC-B) to 100% (CGBAC-A) of *C. gigas* IAP transcripts shared between experiments, compared to 8% (CVBAC-A) to 20% (CVBAC-B) shared between *C. virginica* challenges (Supplementary Table 2). In both species, expression of alternatively spliced versions of the same gene in different challenges accounted for some transcript expression diversity (4 genes in *C. gigas*, 5 genes in *C. virginica*) (e.g. cluster 3, Fig. 6).

Expression patterns of genes with different domain architectures also differed between the two species (Fig. 6). Transcripts from all domain architecture types were differentially expressed to immune challenge in at least one oyster species. No strong patterns emerged regarding specific domain structures or domains associated with particular microbe types (i.e. parasitic, bacterial, or viral). Each experiment, however, expressed a unique assemblage of IAP domain architectures, ranging from 3 (CVBAC-B) to 10 (CVBAC-A) in *C. virginica* and 3 (CGBAC-A) to 11 (CGOSHV1-A susceptible) in *C. gigas* (Fig. 6; Supplementary Table 3). While the DIAP1-like domain architecture was most frequently expressed in *C. virginica* (15 transcripts), the BIRC2/3-like domain architecture was most frequently expressed in *C. gigas* (34 transcripts; Supplementary Table 3). Transcripts containing a UBA domain (cluster 4) were only differentially expressed in response to parasitic challenge in *C. virginica*.

Transcripts containing novel NZBIR (cluster 4), and Type Y (poorly supported group between clusters 2 and 3) domains were only expressed in *C. virginica* challenge experiments (Fig. 6). Novel domain architectures were expressed in response to multiple challenge experiments. The BIRC10 domain architecture (cluster 7) was significantly differentially expressed across all experiments except one *C. virginica* bacterial challenge. BIRC9 (clusters 13, 19) was expressed in both bacterial and viral challenges (Supplementary Table 3). BIRC11 and BIRC12 (clusters 5, 8, 12; and clusters 9, 14, 16, 18, 20 respectively) were expressed in bacterial, viral, and parasitic experiments (Fig. 6).

Constitutively expressed IAP transcripts in *C. virginica* experiments included representatives from 12 of the 14 domain architectures; all except BIRC5-like and BIRC10 (Supplementary Fig. 4; Supplementary Table 3). *C. virginica* and *C. gigas* transcripts from intronless genes were not differentially expressed to any of the immune challenges, though a transcript for one *C. virginica* intronless gene (LOC111132301, BIRC7-like, between cluster 12 and 13) and one *C. gigas* intronless gene (LOC109617982, BIRC11, cluster 12) were constitutively expressed across all experiments (Supplementary Fig. 4). This result indicates that a portion of the full IAP protein diversity may be important in constitutive physiological processes, rather than important during active disease response.

### Oyster annotated genomes possess major apoptosis and regulated cell death pathway proteins, including some from novel cell death pathways

**Fig. 7:**
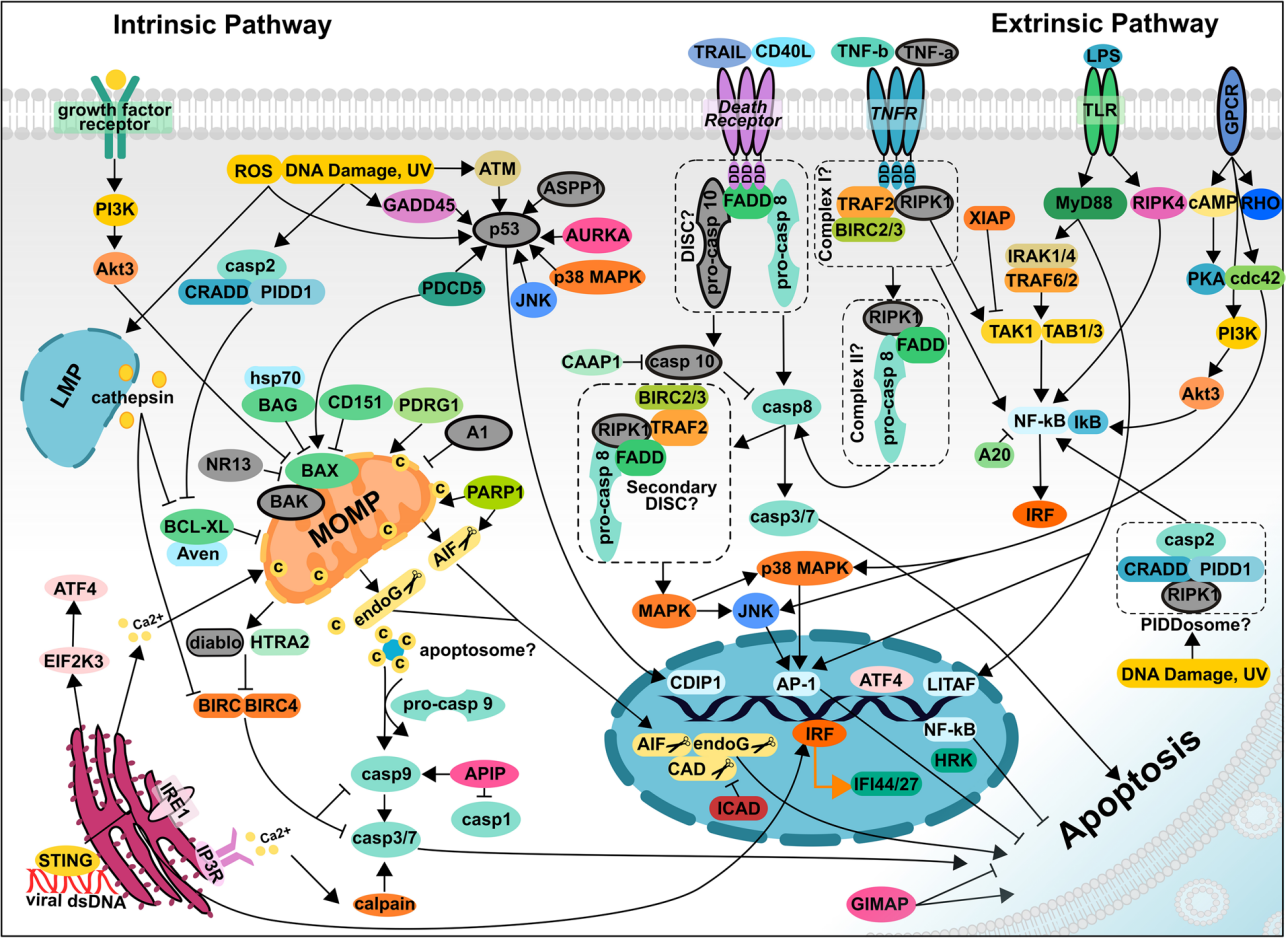
Major intrinsic and extrinsic apoptosis pathway proteins in *Crassostrea virginica* and *C. gigas*. NCBI genome annotations for *C. virginica* (Ostreida) and *C. gigas* (Ostreida) revealed that the major intrinsic and extrinsic apoptosis pathway proteins are annotated in oysters. Proteins present in the *C. gigas* annotated genome and not in the *C. virginica* annotated genome are colored in gray and outlined with a black border. Proteins only in *C. virginica* are in grey. Potential multi-protein complexes are boxed with a dashed black line. Molecules from the novel cell death pathways necroptosis, parthanatos, and lysosome dependent cell death were also identified. Multiple (about 25%) model organism (*D. melanogaster*, *C. elegans*, *H. sapiens*) apoptosis and RCD proteins identified in the literature were not found in oyster annotated reference genomes, either due to errors in annotation or true absence in oysters

To investigate potential relationships between IAP gene expression and apoptotic responses during immune challenge, regulated cell death (RCD) pathway genes and transcripts were identified in *C. gigas* and *C. virginica* annotated reference genomes*,* revealing 1290 unique RCD-related transcripts in *C. virginica* across 676 gene loci, and 844 unique transcripts in *C. gigas* across 511 gene loci (Supplementary Table 4; Additional Files 1 and 2). Key molecules in the intrinsic and extrinsic apoptosis pathways, including receptors, signaling molecules, and effectors, were identified in oyster annotations (Fig. 7). Components of molecular complexes involved in apoptosis were also identified, including the apoptosome (caspase 9, cytochrome c), the PIDDosome (PIDD1, CRADD, casp2, RIPK1), and DISC complexes (RIPK1, FADD, caspase 8, TRAF2).

A few (76 out of 315; 25%) RCD proteins from the literature were absent in oyster reference annotations, due to either low identity with RefSeq proteins, gene loss in genome assembly and annotation, or true absence in oyster genomes. These included mitochondrial apoptosis pathway proteins (BAD, Bcl-w, Bcl-2, BI-1, BID, BIK, BIM, BMF, Bok, Mcl-1, NOXA, HRK, DEBCL, PUMA, Apaf-1, CHOP), and extrinsic apoptosis pathway ligands, receptors, and adapters (FasL and FasR, DR3 (TNFRSF25), DR4 (TNFRSF10A), DR5 (TNFRSF10B), Apo3L (TNFSF12), c-FLIP, TRADD, RIPK3). Cellular tumor antigen p53, diablo homolog, mitochondrial, and Tumor Necrosis Factor (TNF-α) were only annotated in *C. gigas*.

Several proteins involved in regulated cell death pathways other than apoptosis [21] were also annotated, including necroptosis proteins aurora kinase A (AURKA), E3 ubiquitin-protein ligase CHIP (CHIP), protein phosphatase 1B (PPM1B) tumor necrosis factor alpha-induced protein 3 (TNFAIP3), and receptor-interacting protein kinase 1 (RIPK1). Lysosome-dependent cell death cathepsins (cathepsin Z, B, L, L1, O) were identified, as were critical parthanatos proteins poly [ADP-ribose] polymerase 1 (PARP1), hexokinase 1 (HK1), apoptosis inducing factor (AIF, AIFM1), and macrophage migration inhibitory factor (MIF).

### Apoptosis-related gene expression in response to immune challenge

Differential expression of apoptosis-related genes was analyzed for each experiment to determine potential associations between IAP and apoptosis gene expression during immune challenge. The number of apoptosis-related genes differentially expressed in response to immune challenge was much higher in *C. gigas* than *C. virginica* (1632 vs. 440), which could be driven by types of challenge analyzed (e.g. no viral challenge was available for *C. virginica*) and/or differences between the two species in the use of apoptosis (Supplementary Table 5).

**Fig. 8:**
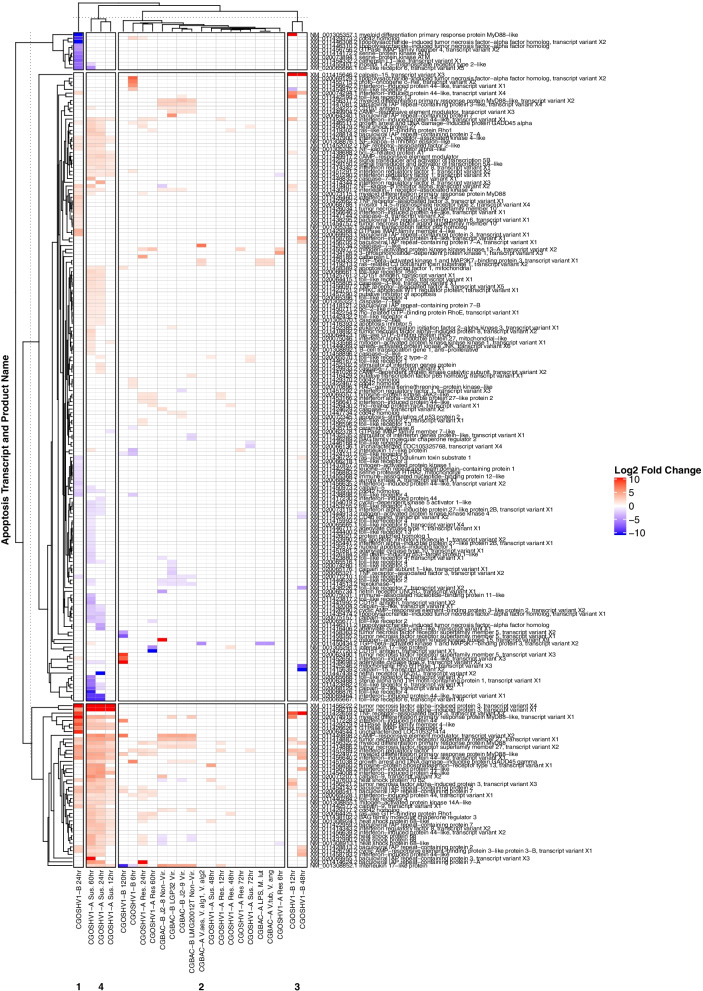
Apoptosis-related *C. gigas* differential gene expression response to immune challenge clustered mainly by susceptibility/resistance. Patterns of apoptosis pathway differential expression in response to each immune challenge in *C. gigas* (Ostreida) were assessed and clustered using a heatmap analysis to assess whether apoptotic responses differed between immune challenge types. This heatmap plots significantly differentially expressed apoptosis pathway transcripts with LFC > 1 in *C. gigas* experimental groups, colored by LFC and generated by ComplexHeatmap. Experimental treatment groups are along the X-axis, clustered by similarity of apoptosis transcript LFC. BAC: bacterial challenge. OSHV1: viral challenge. Sus: susceptible oysters. Res: resistant oysters. Apoptosis transcript IDs followed by their product name assigned by RefSeq are along the Y-axis. Total differentially expressed apoptosis-related transcripts were almost quadrupled in *C. gigas* compared to *C. virginica* (Ostreida). CGOSHV1-A susceptible and CGOSHV1-B experiments showed the most unique patterns of apoptosis expression and strongest extrinsic apoptosis pathway upregulation

**Fig. 9:**
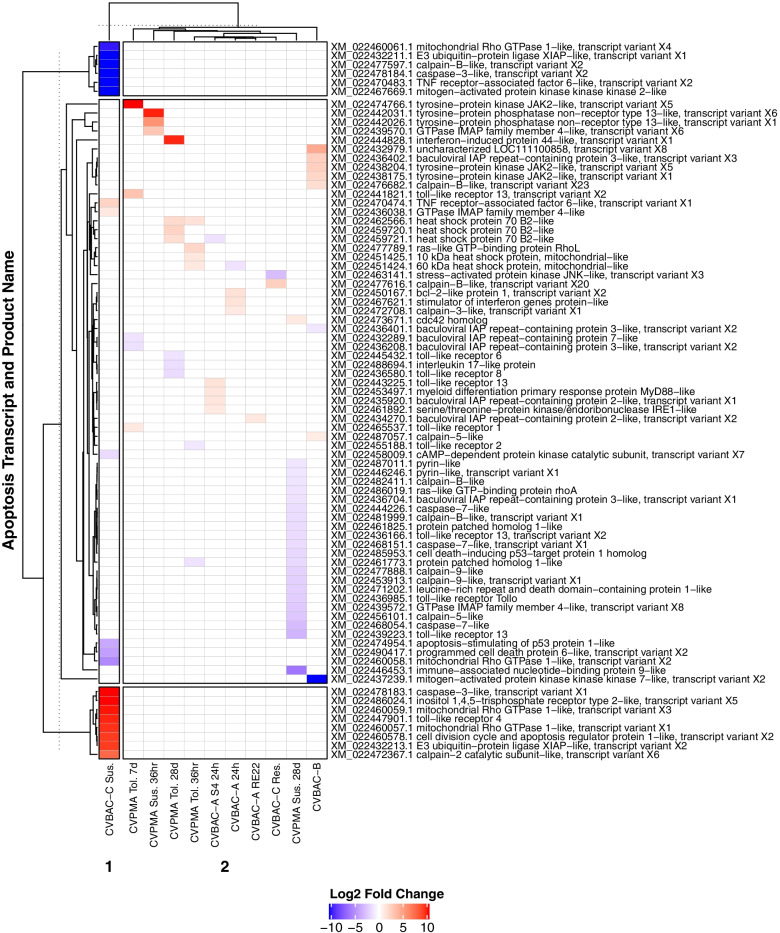
Apoptosis-related *C. virginica* differential gene expression response to immune challenge also clustered mainly by susceptibility/resistance. Patterns of apoptosis pathway differential expression in response to each immune challenge in *C. virginica* (Ostreida) were assessed and clustered using a heatmap analysis to assess whether the apoptotic response differed between immune challenge types. This heatmap plots significantly differentially expressed apoptosis pathway transcripts with LFC > 1 in *C. virginica* (Ostreida) experimental groups, colored by LFC and generated by ComplexHeatmap. Experimental treatment groups are along the X-axis, clustered by similarity of apoptosis transcript LFC. BAC: bacterial challenge. PM*:* parasitic challenge (*Perkinsus marinus*). OSHV1: viral challenge. Sus: susceptible oysters. Res: resistant oysters. Apoptosis transcript IDs followed by their product name assigned by RefSeq are along the Y-axis. CVBAC-C displayed the most unique apoptosis pathway expression, comprised mainly of extrinsic pathway transcripts. CVPMA 28d susceptible oysters also displayed strong downregulation of transcripts involved in apoptosis execution, the TLR pathway, DNA damage response, and mitochondrial dysfunction related proteins

Total apoptosis-related transcripts differentially expressed in *C. virginica* and *C. gigas* immune challenges ranged between 37 (CVBAC-B) and 1040 (CGOSHV1-A) (Supplementary Table 5). Clustering immune challenge experiments by log2 fold change (LFC) in apoptosis-related gene expression showed that levels of susceptibility or resistance (achieved by family-based selective breeding within each oyster host; Table 2) to pathogenic challenge (viral challenge in *C. gigas*; bacterial or parasitic challenge in *C. virginica* [13, 53, 55]) was the strongest factor influencing apoptosis-related gene expression in both host species, with susceptible oysters showing a larger/broader response to challenge than resistant oysters (Figs. 8 and 9)*.* In *C. gigas*, CGOSHV1-A susceptible and CGOSHV1-B oysters showed the most unique apoptosis expression patterns, with strong upregulation of transcripts in the extrinsic, TNFR, and interferon (IFN) pathways (TRAF3, IRF1, MyD88, BIRC3, BIRC7, TNFRSF27, IFI44, FAP1, GIMAP4), and strong downregulation of TLR, mitochondrial apoptosis, and p53 pathway transcripts (TLR2, TLR4, TLR6, SARM1, LITAF, CD151) (Fig. 8). In *C. virginica*, ROD-susceptible oysters (CVBAC-C) had the most unique apoptosis gene expression patterns. These differentially expressed transcripts included several coding for proteins in the extrinsic apoptosis pathway, including those shared with the TNFR and TLR pathways (TRAF6, caspase 3, BIRC4/XIAP, RHOT1, MAP3K2, TLR4, CCAR) (Fig. 9). The *P. marinus* (CVPMA) susceptible 28d oysters also showed downregulation of a large group of apoptosis transcripts involved in apoptosis execution (caspase 7) and the TLR pathway (TLR13, TLR tollo, BIRC3), DNA damage response pathways (PIDD1, CDIP1), and mitochondrial dysfunction related proteases calpains 9, 5, and B (Fig. 9).

### IAP expression of multiple domain architecture types directly correlated with apoptosis gene expression

**Fig. 10:**
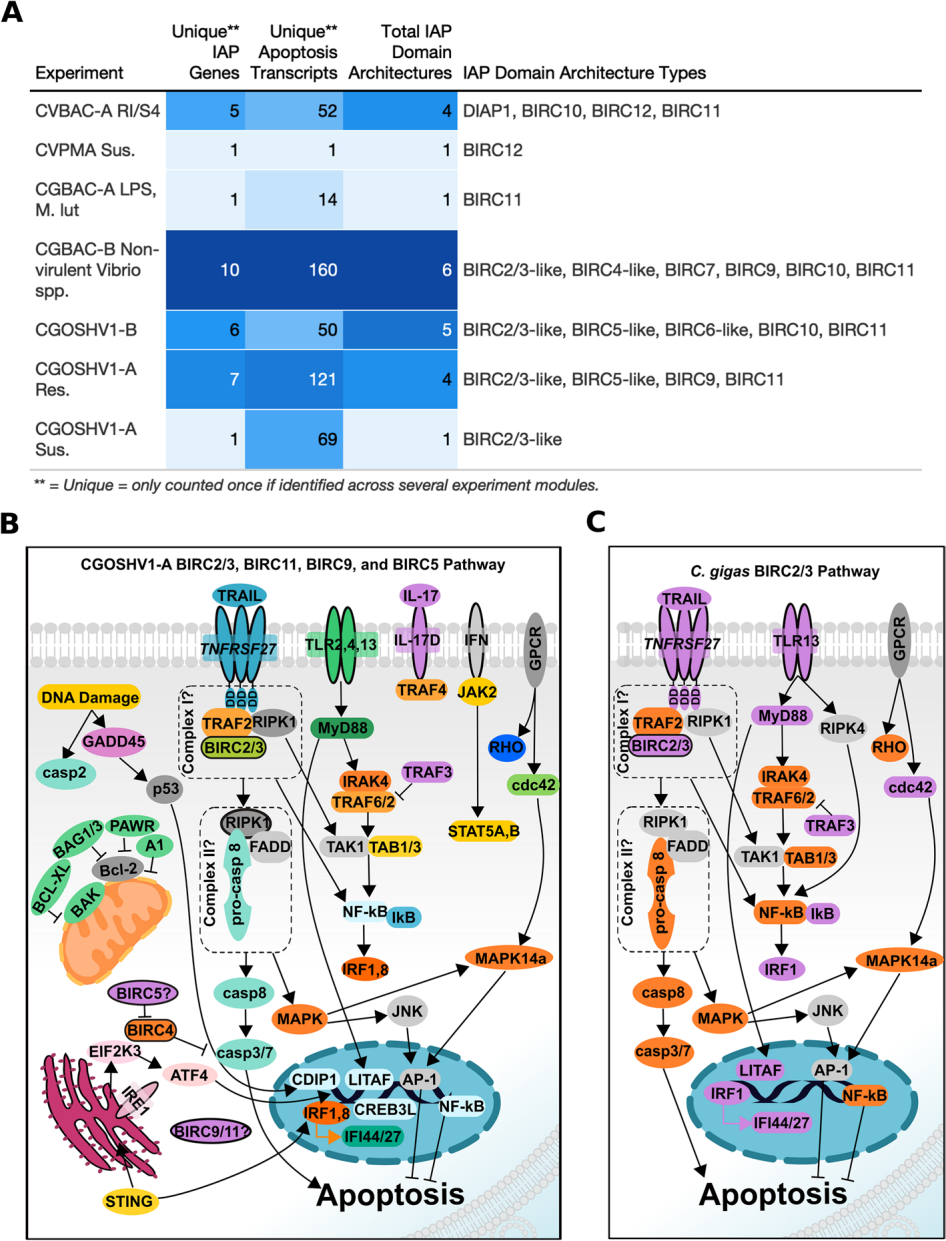
Apoptosis and IAP gene expression in response to immune challenge were directly correlated in oysters. Weighted Gene Correlation Network Analysis (WGCNA) was utilized to determine whether particular IAP domain architectures were associated with particular apoptosis pathways and molecules. **A** Table presenting the number of IAP genes, unique apoptosis transcripts, and domain structures directly correlated in each WGCNA experiment, with darker shading representing higher numbers. The expression of multiple transcripts from a variety of domain architecture types was directly correlated with expression of apoptosis-related transcripts, suggesting the expression of groups of IAPs with potentially different potential functions may be important for apoptosis pathway regulation during immune challenges. **B** Pathway depiction of apoptosis-related transcripts directly correlated with BIRC2/3-like, BIRC5-like, BIRC9, BIRC11 IAPs, as seen in the CGOSHV1-B resistant WGCNA significant modules. **C** Pathway depiction of transcripts in the extrinsic apoptosis pathway whose expression directly correlated with expression of a BIRC2/3-like *C. gigas* (Ostreida) transcript, as seen in the CGOSHV1-B and CGOSHV1-A Resistant experiment WGCNA modules. Purple transcripts were directly correlated in both viral experiments, while those in orange were only directly correlated with this BIRC2/3-like transcript in the CGOSHV1-A Resistant experiment. BIRCs are outlined in black. Molecules outlined in gray were not identified in modules but are important pathway members in selected, well-studied model organisms (*D. melanogaster*, *H. sapiens*, *C. elegans*)

Expansion of the IAP gene family in oysters may have allowed for evolution of new functions, including nuanced control of diverse apoptotic pathways or other functions not related to regulation of apoptosis. To determine whether specific IAP domain architectures were associated with specific apoptosis-related pathways or genes, WGCNA was performed (Fig. 10a,b, Supplementary Tables 6 and 7). In *C. virginica,* expression of IAPs with multiple domain architectures in response to multiple disease challenges was directly corelated with apoptosis genes. In larval oysters exposed to probionts RI and S4 (CVBAC experiment), 5 IAP genes with 4 domain architectures correlated with 52 unique apoptosis-related transcripts. In susceptible oysters exposed to *P. marinus* (CVPMA experiment), one IAP gene identified as BIRC12 correlated with a caspase 7-like transcript (Fig. 10a). In *C. gigas*, IAPs with multiple domain architectures were directly correlated with apoptosis-related transcripts in several experiments, and the CGOSHV1-A resistant and CGBAC-B experiments had the highest number of apoptosis-related transcripts correlated with IAP expression (Fig. 10a).

At least one transcript from each of the domain types, with the exception of BIRC2/3 – NZBIR, was directly correlated with apoptosis-related genes in both oyster species. Multiple unique IAP domain architecture types across modules were directly correlated with apoptosis-related transcript expression in most experiments (CVBAC-A, CGBAC-B, CGOSHV1-B, CGOSHV-1 A Res.) (Fig. 10a). Transcripts from multiple domain architectures were also expressed in the same modules during bacterial and/or viral challenge (Fig. 10a), suggesting IAP domain architectures are not specific to particular immune challenge types and that different domain architectures may work together or have complementary functions. For example, in Pacific oysters exposed to OsHV-1 [55], BIRC2/3, BIRC11, BIRC9, and BIRC5 showed direct correlation with genes in the extrinsic apoptosis/TLR pathway, inflammation, mitochondrial apoptosis (e.g. BAG, BAK, Bcl-xL), antiviral responses (e.g. IFIs, IRFs, IL17RD, JAK, STAT, STING), necroptosis (CHIP, PPM1B), ER stress (ATF-4, EIF2K3, CREB3Ls), executioner caspase 7, and DNA damage response caspase 2 (Fig. 10b, Supplementary Fig. 5). These results demonstrate a complex set of pathways are activated in Pacific oysters in response to viral challenge, and that novel BIRCs may have complementary roles in these pathways (Fig. 10b).

Expression of transcripts for the BIRC2/3-like IAP domain architecture was directly correlated with expression of apoptosis-related transcripts in all *C. gigas* experiments except CGBAC-A, suggesting a consistent association of this transcript with apoptosis in this species. Specifically, *C. gigas* BIRC2/3-like transcript XM_020068541.1 (LOC105331304) was consistently associated with TNFRSF27, TNFSF10 (Apo2L), downstream ISGs and IRFs, and the TLR13 pathway (Fig. 10c). Expression of this transcript was also correlated with expression of transcripts for caspases 1 and 6 and TRAF3 (Fig. 10c). Association of this transcript with the TNFR and IFN pathways and direct correlation with TRAF3 suggest it may have similar signal adapter functions to mammalian BIRC2/3 [24, 56].

Finally, potential patterns of IAP domain architecture co-expression with specific apoptosis pathways or genes was assessed by clustering the direct correlations in each experiment by presence (red) or absence (blue) using a heatmap (Supplementary Fig. 5). Similar to what was observed in Figs. 8 and 9, patterns of directly correlated IAP domain architectures and apoptosis pathway transcripts identified in the WGCNA clustered mostly by experiment and not by domain architecture type (Supplementary Table 3).

## Discussion

Recent whole genome sequencing of marine invertebrates has revealed large scale expansions of immune gene families, including several related to regulated cell death [3, 4, 6, 8, 57–62]. Functional diversification of expanded immune gene repertoires may contribute to the remarkable ability of invertebrates to mount specific responses to immune challenge in the absence of traditional adaptive immunity [3, 6, 37, 61]. Using a comparative genomic and transcriptomic approach, this research: 1) Described great IAP expansion and diversity in oysters, with mechanisms like mutation, tandem duplication, and retroposition leading to novel domains and domain architectures that may allow for unique functionality; 2) Showed that each oyster species expressed unique and variable assemblages of IAP genes and domain architectures in response to immune challenges; 3) Annotated regulated cell death proteins in the genomes of two oyster species, *C. gigas* and *C. virginica*, that had not been previously recognized; and 4) Revealed direct correlation of diverse oyster IAP assemblages with apoptosis pathways across different immune challenges, with levels of resistance to pathogenic challenge effecting apoptosis-related gene expression in both oyster species. These results suggest a role for the expanded IAP family in regulating complex cell death pathway responses to a variety of immune challenges.

### Mechanisms of IAP lineage specific expansion in oysters include tandem duplication and retroposition

As shown in previous research [6], IAP gene expansion differs considerably across molluscs, ranging from 10 genes in *O. sinensis* to 88 in *B. glabrata*, suggesting divergent evolutionary rates and/or selection pressures. Recent investigation of tandemly duplicated IAP genes in the hard clam, *Mercenaria mercenaria,* suggested that IAPs may evolve by purifying selection following duplication [6]. As in *M. mercenaria,* tandem duplication of IAP genes is likely a predominant gene family expansion mechanism in *C. virginica*, (and likely in *C. gigas*) with the majority of IAP genes in *C. virginica* (54 genes, 78% of the IAPs) present in large tandemly duplicated clusters on chromosomes 6 and 7. Tandem duplication as a mechanism of IAP gene family expansion in *C. gigas* has also been noted in the literature [63]. Moreover, tandem duplication as an immune gene expansion mechanism has been noted for other oyster immune gene families, including TNF, MyD88, TLR, Hsp70, and C1qDC [64–68]. The larger repertoire of IAP genes in *C. virginica* compared to *C. gigas* may be due to differences in evolutionary pressure, leading to an increased number of tandem duplications in eastern oysters, and/or potential gene loss in *C. gigas* over time. Further investigation of differences in evolutionary rates and history is necessary to make a conclusion regarding overall IAP gene family evolution in these two species. The recent availability of chromosome-based assemblies for *C. gigas* will facilitate this analysis (GCA_902806645.1, cgigas_uk_roslin_v1) [69].

Retroposition is another prominent mechanism of gene family expansion [17]. Gene retroposition involves insertion of DNA sequence into a genome in a different location from the parent gene following reverse transcription from mRNA. These genes typically lack introns and other regulatory sequences, though retrogenes are transcribed and functional in some cases [70]. Retroposition as a mechanism of gene expansion has been noted for several immune gene families in molluscs, including the IAP family in *M. mercenaria,* the IL-17 family and fibrinogen-related proteins (FREPs) in *B. glabrata*, and IκB genes in *C. gigas* [44, 71, 72]. The number of intronless IAP genes (suggesting retroposition) detected in this research varied across targeted species and intronless IAPs comprised a fewer percentage of total IAPs in both *C. gigas* and *C. virginica* than the hard clam *M. mercenaria* (3 in *C. virginica*, 7 in *C. gigas,* and 51 in *M. mercenaria* [6]). Domain analysis of *C. virginica* IAPs revealed several genes with machinery for both LTR and non-LTR type retroposition in translated IAP ORFs, providing further support for past retroposition in this family.

Interestingly, intronless *C. virginica* IAPs lacked retroposition machinery, suggesting they could be retroposed copies from a parent gene that are no longer active retrotransposons, or could be active retrotransposons by relying on machinery from other genes [70]. Intronless IAPs in both *C. virginica* and *M. mercenaria* may retain some functionality, with several *M. mercenaria* IAPs noted to have high expression levels in response to environmental stress [6] and one *C. virginica* IAP constitutively expressed to immune challenge in this research. Overall, this research indicates that tandem duplication is the predominant mechanism of *C. virginica* IAP expansion but that retroposition may still play an important role.

### IAP expansion in oysters allowed for evolution of novel BIR domain sequences and domain architectures

Humans possess 8 known IAPs, while *Drosophila* spp. possess 2 [24], and each contains a distinct assemblage of domains which confer unique functions [46]. Interproscan functional analysis revealed IAPs in oysters have greater structural domain architecture diversity than mammals and fruit flies, with 14 total domain architecture types identified, including 8 types with architectures similar to human or fruit fly IAPs and 4 novel types (Fig. 5). The only mammalian IAPs without a similar IAP in oysters were BIRC1 (NAIP) and BIRC8 (ILP2). Domain architecture types in oysters varied in number of BIR repeats, the type of BIR domain (including three novel BIR domain types; X,Y, and NZBIR, see below) and the presence or absence of domains characteristic of IAPs; RING domains, DD instead of CARD, UBA and UBC domains, suggesting a complex history of domain loss and gain over time that may have involved parallel evolution or retention of ancestral forms from a common ancestor [6, 73].

Interestingly, BIRC2/3 IAPs, similar to other molluscan IAPs [63], lacked the CARD domain characteristic of mammalian IAPs, possessing instead a DD (BIRC10 and BIRC11 also possessed a DD as well). Despite lacking true CARD domains, the presence of DDs in these oyster IAPs may still allow for mediation of key protein-protein interactions during apoptosis. DD and CARD domains are structurally similar and both mediate protein-protein interactions critical in apoptosis transduction [74]. In mammalian BIRC2/3, the CARD domain promotes protein stability by preventing RING-domain meditated auto-ubiquitination [75]. During intrinsic apoptosis, a CARD-CARD interaction between Apaf-1 and caspase 9 allows for caspase 9 activation [76]. DD-containing proteins in *D. melanogaster* have also been shown to complex with caspase molecules, and in mammals formation of the PIDDosome during DNA-damage response involves DD-containing proteins PIDD and CRADD complexing with caspase-2 [77, 78]. WGCNA analysis in this research revealed direct correlation between DD-containing IAPs and caspase expression, suggesting DD-containing oyster IAPs could potentially function similarly to CARD domains. The ability of DD-containing oyster IAPs to directly interact with other apoptosis proteins, such as caspases, should be investigated in the future.

Expansion of novel IAP domain architectures in oysters is also supported by a recent study of *M. mercenaria* IAPs [6]. In the hard clam, 9 distinct architectures were identified and all but two (classified as Type D and E) were also identified in oysters [6]. However, Song et al. (2021) did not consider BIR Type or the presence of UBA or DD domains in clam IAP characterization [6]. Though all types identified in this oyster study were identified in the *M. mercenaria* study, inclusion of these additional domains in the present analysis gave our work the ability to distinguish between expression patterns of novel types and model organism types, such as BIRC10 (TII-DD), which was combined with BIRC5-like proteins (TII) in the *M. mercenaria* G1 type, and BIRC11 (TII-DD-RING or BIR*-DD-RING) which was combined with BIRC7-like (TII-RING) in the *M. mercenaria* C type [6]. The functionality of these novel types, in addition to conserved model organism types, supports the utility of IAP expansion in allowing for functional diversification.

Despite high levels of lineage specific IAP expansion in molluscs, phylogenetic analysis of IAP amino acid sequences revealed that all BIRC5-like and BIRC6-like proteins are highly related between molluscan species, suggesting functional conservation of these sequences over evolutionary time (Fig. 1b). Both BIRC5 and BIRC6 play important apoptosis regulatory roles in mammals, but BIRC5 (Survivin) is also essential for cell division [79], while BIRC6 (BRUCE) proteins play critical roles in mitosis, autophagosome/lysosome fusion, DNA double strand break repair and DNA replication [32, 36]. Performance of these critical cell cycle and cell division functions may have constrained their sequence evolution and led to low divergence over evolutionary time as compared to other IAP proteins.

BIR domains are the critical functional domain of IAPs and are traditionally classified as Type I or Type II, with Type II BIRs able to physically interact with IAP-binding motif (IBM) containing proteins smac/DIABLO or caspases [46]. Analysis of BIR domain sequences revealed oysters possess both model organism Type I and Type II repeats, as well as divergent types named here Type X, Type Y, and NZBIR (not found in any other organism in the NCBI database, based on *blastp*). Conserved Type II domains, likely able to interact with IBM-containing proteins based on sequence analysis [80], were the most prominent across oyster BIRs (62% of all BIR domains in *C. virginica*, 66% in *C. gigas*). Consistent with this hypothesis, WGCNA analysis indicated direct co-expression of caspases with IAPs possessing Type II repeats (Supplementary Fig. 5). Moreover, a previous functional study of an IAP in *C. gigas* (LOC1053280490), classified in this paper as BIRC2/3-like, found its Type II BIR2 repeat was able to mediate interaction with caspase 2 [81].

Several oyster IAP genes (BIRC2/3-like and BIRC9, Fig. 4) contained novel BIR types (Types X, Y, and NZBIR) in addition to at least one Type II BIR. Proteins containing novel oyster BIR types were distributed across the IAP phylogenetic tree, suggesting that they may have arisen due to mutations in tandemly duplicated genes independently in *C. virginica* and *C. gigas* (Supplementary Fig. 3a)*.* It is not known if oyster IAPs with these novel domains are functional, either as IAPs or other novel functions, but genes containing each novel BIR domain were significantly differentially expressed in response to immune challenge and co-expressed with apoptosis-related genes in at least one oyster species (more on this in sections below). The presence of at least one Type II BIR in these novel oyster IAPs should preserve their ability to interact with IBMs. The N-terminal BIR Type I repeat in mammalian BIRC2, which is replaced in the novel oyster BIRC2/3-like IAPs by an NZBIR type, is necessary and sufficient for binding to SMAC and TRAF2 [82]. Though NZBIR-containing BIRC2/3-like proteins contain a Type II BIR and a UBA domain similar to mammalian BIRC2/3, lack of a third BIR domain and/or alteration of the N-terminal BIR domain may affect this critical function of BIRC2/3 like proteins. While these genes are expressed in *C. virginica,* lack of significant differential expression of NZBIR and Type Y containing IAPs in *C. gigas* suggests these transcripts may respond to other types of environmental or immune challenges in *C. gigas*, or are non-functional. Functional studies should evaluate the potential contributions of these novel BIR domains to IAP function and identify their potential interaction partners.

### Eastern and Pacific oysters expressed diverse IAP domain architecture repertoires in response to immune challenge

Overall IAP gene usage in oysters in response to diverse immune challenges (Table 2) was investigated in this research. Most (77% of *C. virginica* and 82% of *C. gigas*) IAP genes were differentially or constitutively expressed in response to one or more challenges, suggesting that most of the expanded IAPs are functional and involved in immunity. It is possible that IAP genes not expressed in these challenges respond to other stressors and/or at life stages not assessed in this study. For example, *M. mercenaria* IAPs were strongly responsive to challenge with aerial exposure, low salinity, high temperature, or low oxygen, revealing IAPs may play important roles in response to both environmental and disease challenge [6].

Interestingly, *C. virginica* largely expressed different gene sets between challenge experiments, while *C. gigas* more often expressed overlapping gene sets to different challenges, suggesting that greater IAP expansion may allow for greater specificity of IAP gene usage in response to different challenges in *C. virginica*. These results should be interpreted with caution, however, since sampled experiments were performed in diverse experimental conditions with oysters at different live stages (from larvae to adults), and with sequencing performed for both oyster pools (larval experiments) and single individuals. Comparative analysis between IAP responses to immune challenge in these two species was also restricted because both are affected by different diseases (consistent with their different geographical distribution [11]), and no transcriptome experiments were currently available at the time of this research in which both species had been concurrently challenged with the same pathogen at the same developmental stage [11]. Finally, natural infection with OsHV-1 in *C. gigas* typically involves co-infection with *Vibrio* spp. which may contribute to strong similarities in IAP and apoptosis pathway responses between natural OsHV-1 exposure (CGOSHV1-A) and *Vibrio* spp. experiments [55]. Future challenge experiments of both species using the same pathogens and pathogen associated molecular patterns (PAMPs) such as bacterial LPS and the viral response stimulator poly(I:C) [83, 84] would allow for better determination of differences in IAP usage between the two species.

Next, analysis of IAP domain architecture expression in oysters revealed expressed IAP genes in both species were from multiple domain architecture types, and all domain architecture types, including novel types, were significantly differentially expressed in at least one challenge. None of the domain architecture types appear to be specific to challenge type (parasitic, bacterial, or viral). The domain architecture most frequently differentially expressed in *C. virginica* was the DIAP1-like, while in *C. gigas*, it was the BIRC2/3-like. WGCNA analysis next indicated significant correlation between several domain architectures in immune challenges, suggesting multiple IAPs with different putative functions may function in the same pathways or participate in different pathways that are co-regulated during immune challenge (Fig. 10a). However, the expression of unique assemblages of IAP domain architectures in response to the different challenges also suggests that overall IAP activity can be tailored to specific situations. These results support that the expanded IAP genes and domain architecture types in oysters are not merely non-functional artifacts of duplication events and domain loss and gain but allow for critical tailoring of immune responses, which has been previously shown for other expanded gene families such as TLRs and NOD-Like Receptors [85].

### IAP expression was directly correlated with apoptosis gene expression suggesting roles in finely regulating apoptosis during immune challenge

Expression of a variety of RCD pathways, including intrinsic and extrinsic apoptosis, parthanatos and necroptosis, differed between challenge type and species. Consistent with known roles of apoptosis in immune response and disease in a variety of organisms, including oysters [52, 86, 87], viral challenge in *C. gigas* elicited the strongest apoptotic response, while probiotic challenge in *C. virginica* elicited the weakest apoptotic response. Interestingly, the assemblage of expressed IAP and apoptotic transcripts was affected most strongly by the host’s susceptibility to particular challenges, with eastern oysters susceptible to *Aliiroseovarius crassostreae* (CVBAC-C) and Pacific oysters susceptible to viral challenge (CGOSHV1-A) showing the largest changes in gene expression (Figs. 8 and Fig. 9). These results are consistent with previous functional research suggesting a role of apoptosis in disease susceptibility (or resistance) in oysters and other species [18, 53, 88–92]. Network analysis additionally revealed that viral exposure experiments in *C. gigas* [13, 55] showed the highest diversity of IAP domain architecture transcripts, (BIRC2/3-like, BIRC5-like, BIRC6-like, BIRC10, and BIRC11) directly correlated with expression of transcripts in multiple RCD-related pathways (extrinsic and mitochondrial apoptosis, inflammation, antiviral response, necroptosis, and ER stress).

Multiple IAP domain architecture types were directly correlated with apoptosis-related transcripts across experiments, including novel IAP domain architectures (BIRC9, BIRC10, BIRC11, BIRC12), and the combination of expressed IAP domain architecture types differed between each experiment. This result suggests that the importance of IAP expansion in oysters is to allow for expression of multiple IAPs of different potential functional types to fine tune regulation of apoptotic responses to various immune challenges. Expression of an assemblage of IAPs may also provide redundancy and extra safeguards against aberrant apoptosis. In WGCNA networks, expression of many IAPs was also directly correlated with expression of other IAP domain architecture types, suggesting they may be co-regulated, interact with one another in the same apoptosis pathway, be part of dually activated regulated cell death pathways, or be involved in crosstalk between multiple apoptosis pathways. Indeed, in humans, IAPs have demonstrated the ability to perform in concert and form IAP-IAP complexes, with BIRC5 (survivin) specifically forming a complex with BIRC4 (XIAP) [93]. Moreover, crosstalk between IAPs in mammals has been previously shown to affect IAP levels [93–96]. These results together support that rather than individual IAP domain architecture types being associated with single apoptosis pathways or immune challenge types, IAP expansion has allowed for expression of an orchestrated collection of diverse IAPs in order to tailor an apoptosis regulatory response to unique challenges.

Analysis of IAP transcripts directly correlated with apoptosis pathway transcripts across multiple experiments also allowed for identification of a novel *C. gigas* BIRC2/3-like transcript, XM_020068541.1 (LOC105331304) which may have homologous function to BIRC2/3 in mammals (Fig. 10c). This transcript showed similar domain architecture to mammalian BIRC2/3, though with a DD instead of CARD, and in *C. gigas* was directly correlated with extrinsic pathway partners similar to mammalian BIRC2/3, including TNFR and IFN pathways and direct correlation with TRAF3 [24, 56]. In mammals, BIRC2/3 proteins are ubiquitin ligases involved in TNFR signaling and activation of the NF-κB pathway [97]. In addition to assessing the ability of this protein and other oyster BIRC2/3-like proteins to perform E3-ubiquitin-ligase activity, future functional studies should assess the potential for expanded oyster BIRC2/3-like proteins to interact with different members of the expanded oyster TNFR and TRAF families [3].

### Oysters contain novel regulated cell death pathway components

To determine the potential role of IAPs in RCD, this research performed an in-depth identification of apoptosis and regulated cell death molecules present in *C. virginica* and *C. gigas*, confirming, updating, and expanding molecules identified in previous studies [6, 14, 19, 20, 72, 98–102]. It also provided an updated list of RCD-related genes for further work. Lack of annotation of certain oyster apoptosis transcripts present in model organisms should be investigated in-depth using manual annotation methods to determine whether these are truly absent in these oysters or were not annotated due to low sequence identity or limitations in an annotation approach relying on RefSeq assigned annotations. For example, while cellular tumor antigen p53 was not explicitly annotated in the *C. gigas* reference genome utilized, previous studies using manual annotation approaches have identified p53 homologs in *C. gigas* and demonstrated the involvement of *Cg-p53* in mitochondrial apoptosis [98, 103]. P53 has also been previously identified in other molluscs, including *Mytilus galloprovincialis,* the soft shell clam *Mya arenaria,* and the blue mussel *Mytilus edulis* [99, 104]. Previous manual annotation approaches have also recognized Bcl-2 family homologs in *C. gigas* including *Cg-Bcl2* (not annotated in the reference), *Cg-Bcl-xl* (present in annotation), *Cg-Bak* and *Cg-Bax* (present in annotation), and demonstrated their role in apoptosis regulation in a yeast *Saccharomyces cerevisiae* model [98, 100]. Members of the BH3-only Bcl-2 family of proteins, including BIK, BID, BIM, BAD, PUMA, NOXA, and HRK, have yet to be identified in molluscs [98, 100].

To our knowledge, this is the most in-depth description of novel regulated cell death pathway molecular components in oysters and this research identified proteins involved in necroptosis, lysosome-dependent cell death, and parthanatos. Molecules involved in parthanatos, including PARP1, and MIF have not been previously discussed in molluscs, while AIF, which is involved in caspase-independent apoptosis, has been previously recognized in several species [100]. Isolated necroptosis pathway components, however, have been previously identified in oysters and molluscs. First, the mitochondrial serine/threonine protein phosphatase PGAM5, which is involved in inflammasome activation and operates downstream of RIPK3 during necroptosis, has been identified in *C. gigas* mitochondria in response to hypoxia and reoxygenation stress [105]. Assessment of the transcriptional response of warm acclimated abalone *Haliotis rufescens* has previously revealed regulation of the necroptotic process [106]. Additionally, in the oyster *Crassostrea hongkongensis*, TRAF6 was found to suppress apoptosis through activation of the necroptosis regulatory protein pellino, which is known to regulate ubiquitination of RIPK1, a key necroptosis enzyme [107]. TNFAIP3 was additionally identified as a potential target for neurotransmitter-responsive miRNAs in *C. gigas* and has been shown to respond to thermal and low salinity stress in the Sydney rock oyster *Saccostrea glomerata* [108, 109]. Finally, RIPK1 has been previously recognized in *Lingula anatina*, and in *Octopus maya* under chronic thermal stress [110, 111]. These results together support that the necroptosis pathway may be found across molluscs and play diverse roles in environmental stress response.

## Conclusion

This research used a genomic and transcriptomic approach as a first step in the characterization of the role of IAP gene expansion in oyster apoptotic response to immune challenge. It also offers an updated and expanded characterization of the apoptotic pathway in oysters and demonstrates the power of a novel, cross-species comparative transcriptomic approach to investigate the potential role of expanded immune gene families in invertebrate immune response. Using this approach, we revealed substantial diversity in the IAP family at the level of genes, BIR domains, and domain architecture that were expressed during immune challenge. Domain variation across IAP domain architectures in molluscs likely resulted from a complex history of domain loss and gain over time.

This research also demonstrated direct correlation of IAP gene expression with expression of apoptosis-related genes. Usage of a different assemblage of IAP genes and domain architecture types in apoptosis pathways across experiments may allow for unique regulation of apoptosis proteins that cannot be understood until further functional work is performed to assess novel BIR domain and domain architecture types. This research suggests that lineage specific expansion in the number of IAP genes in oysters has allowed for the development of novel domain architecture types which may confer uniquely tailored apoptotic responses to immune challenge. Overall, this research represents major steps toward fully characterizing the molecular machinery of apoptosis and regulated-cell death pathways in oysters and understanding the role that diversified and expanded IAPs may play in apoptosis regulation, and provides further evidence that gene expansion is a critical mechanism allowing invertebrates to mount diverse immune responses to disease.

## Methods

### IAP gene family identification and phylogenetic analysis

Annotated molluscan genomes [10] across multiple classes were retrieved from NCBI for IAP gene family identification: the California sea hare *Aplysia californica* (Heterobranchia), marsh snail *Biomphalaria glabrata* (Heterobranchia), eastern emerald elysia *Elysia chlorotica* (Heterobranchia), Pacific oyster *Crassostrea gigas* (Ostreida), eastern oyster *Crassostrea virginica* (Ostreida), owl limpet *Lottia gigantea* (Patellogastroda), yesso scallop *Mizuhopecten yessoensis* (Pectinida), California two spot octopus *Octopus bimaculoides* (Octopoda), east Asian common octopus *Octopus vulgaris* (*sinensis*) (Octopoda), and golden apple snail *Pomacea canaliculata* (Coengastropoda) (Table 1). This broad array of molluscs across several classes was chosen in order to provide a broader basis for comparison between IAP gene expansion in oysters (*C. virginica* and *C. gigas*) and other classes. Specific genomes in each class were selected from those available at the time of the analysis based on overall genome completeness and quality.

Rather than relying on the protein annotations identified by prior software and made available for these genomes on NCBI, which may mis-annotate expanded gene family sequences even in well-studied species [112], IAPs were identified by the presence of their defining BIR domain from the protein sequences themselves using two computational methods, HMMer (V 3.2.1) [113, 114] and Interproscan (V 5.44) [115]. First, the HMMbuild tool created a hidden Markov model (HMM) from a list of model organism BIR sequences compiled from the curated Pfam (V 32.0) BIR domain model (PF00653). The HMM was compared against the protein annotation for each species with the HMMsearch tool. Putative IAP protein sequences (E-value < 0.001) were further analyzed with Interproscan to identify functional domains [115]. Those lacking a BIR repeat signature as identified by the Conserved Domain Database (CDD, search included in Interproscan analysis) were removed and exact duplicates in protein coding sequence were collapsed with CD-HIT for downstream analysis [116]. Redundant *C. virginica* IAP sequences caused by genome assembly artifacts (haplotigs) (Puritz et al. *in prep*) were also removed (Supplementary Table 8). To do this, alignments of IAP protein sequences were built with MAFFT (V 7.45; auto setting) [49, 116] and visualized in Uniprot UGENE [117]. Protein sequences in clusters with > 95% similarity showing lower raw read mapping coverage (< half coverage compared to other proteins in the cluster as identified with CD-HIT) were suspected as haplotigs and removed from further analysis (Supplementary Table 8). In the RNAseq analysis, read counts from suspected haplotigs were added to the counts for their “parent”.

Phylogenetic trees of molluscan or bivalve IAP amino acid sequences were built using RAxML HPC MPI (V 8.2.1) [115, 116] with the model PROTGAMMAAUTO, and performing rapid bootstrap analysis and maximum likelihood tree searching using the `autoMRE` bootstrap convergence criterion [118, 119]. *Octopus* spp. (*O. bimaculoides*, *O. sinensis*) and scallop (*M. yessoensis*) were used as outgroups for the molluscan and bivalve trees, respectively, in accordance with previous literature [61, 68]. Phylogenetic trees were generated with ggtree [120] and protein domains were visualized using ggplot `geom_segment` and compiled with cowplot (V 1.0.0, Wilke, Claus). Chromosomal locations of IAP genes in the *C. virginica* genome assembly (V 3.0 GCA_002022765.4) were plotted using Rcircos (V 1.2.1) [121]. Intronless genes were identified as genes with a single exon in the annotation “gff3” file for both *C. virginica* and *C. gigas*.

### BIR domain classification and IAP domain architecture analysis

Oyster BIR domains identified by the CDD and Interproscan search were classified by aligning the oyster sequences to BIR domain amino acid sequences from well-studied model organisms across a range of taxa (*D. melanogaster*, *Homo sapiens*, *Mus musculus, Danio rerio*) using MAFFT (V 7.45; setting `-auto` (BIR domain Multiple Sequence Alignment, Additional File 4) and viewed in UGENE for analysis [117]. Phylogenetic analyses of BIR domains were performed and visualized as described above using MAFFT and RAxML. Sequences were categorized according to sequence patterns in the α-3 and α-4 sequence regions and amino acid identities at critical positions. Type I and Type II classification corresponded with conservation of critical amino acids between oyster and model organism sequences [46, 47, 122]. Oyster sequences lacking conserved amino acids at key positions were considered novel types and were further characterized by the amino acid properties at these key locations (hydrophobic, hydrophilic). Secondary protein structure prediction of BIR domains was performed using RaptorX with auto settings [123]. Three class secondary structure (H = alpha helix, E = beta sheet, and C = coil), and eight class secondary structure (H = alpha helix, G = five turn helix, I = extended strand in beta ladder, E = isolated beta bridge, T = hydrogen bonded turn, S = bend, L = loop) were determined for each BIR amino acid position [123].

Additional functional domains were identified in mollusc IAP amino acid sequences during the initial Interproscan analysis. IAP sequences from *C. virginica* and *C. gigas* were clustered into functional domain architecture groupings based on BIR domain patterns (number and type of BIR domains), the presence of RING finger domains, Death Domains (DD), UBA domains, bootstrapping support in the RAxML tree (> 90%), and presence of both *C. virginica* and *C. gigas* proteins in the cluster. Domain architecture structures were compared to model organisms *D. melanogaster* and *H. sapiens* where IAP domain organization and function has been very well characterized, and oyster IAP domain architectures not found in these model organisms were considered novel [26, 51].

### Identification of apoptosis and regulated cell death genes in *C. virginica* and *C. gigas*

A list of candidate apoptosis and regulated cell death proteins previously identified in selected model organisms (*D. melanogaster*, *H. sapiens*, *C. elegans*) and molluscs was gathered via literature search and the Kyoto Encyclopedia of Genes and Genomes (KEGG) reference apoptosis pathway [3, 4, 8, 13, 19, 20, 58, 59, 124–128]. UniprotKB was used to identify known protein aliases for each protein [129]. Eastern oyster (V 3.0, GCA_002022765.4) and Pacific oyster (V 9.0, GCA_000297895.1) reference genome annotations were mined for protein names and aliases in the target list using R (V 3.6.1).

### Oyster transcriptomes in response to immune challenge

Apoptosis gene expression was compared across four distinct challenge types (viral, bacterial, parasitic, and probiotic) in two species (*C. virginica* and *C. gigas*) and 8 transcriptome experiments, containing 199 total raw transcriptomes spanning a variety of conditions (Table 2; Supplementary Table 9). Raw transcriptome data was downloaded between 2016 and 2020 from the NCBI SRA database using the SRA Toolkit (V 2.9.0) [130]. BBTools BBMap (V 37.36) was used to trim adapters, quality trim the left and right sides of reads with Phred quality scores of less than 20, and remove entire reads with an average Phred score of less than 10 [131]. Transcriptomes were aligned to their respective NCBI reference genome sequences using HISAT2 (V 2.1.0) with default parameters and without use of a reference annotation to allow for novel transcript discovery [132, 133]. HISAT2 output files were sorted and converted into BAM format using SAMtools (V 1.9.0) [134]. Transcripts were assembled and quantified for each experiment separately using their respective reference genome annotations (Table 1) using Stringtie (V 2.1.0) [133]. Comparison of transcriptome annotation to the reference for each sample was conducted using gffcompare (V 0.11.5) [133]. Stringtie output was formatted into matrices of transcript count data and uploaded into R Studio (V 3.6.1) [135].

### Gene expression analysis

Differential transcript expression was calculated for each experiment separately using the package DESeq2 (V 1.24.0) [136] (Table 2). Models were designed for each experiment to determine the overall effect of immune challenge. Experiments with multiple experimental conditions or timepoints were split so that specific effects in each experimental condition (e.g. time after challenge, host genetics and age) could be measured. In experiments lacking either controls or replicates for each condition, the effect of condition was corrected in the DESeq model design by pooling similar conditions (Supplementary Table 10).

Transcripts with < 10 read counts were removed from analysis. Log fold change (LFC) in expression between genes within experiments were considered significant when *p*-values adjusted (*Padj*) using the Benjamini–Hochberg to control for the False Discovery rate (FDR) were ≤ 0.05. LFC shrinkage was performed using “apeglm” to improve ranking genes by effect size and enable comparison of LFC between experiments [137]. Transcript counts were log scale transformed and normalized to the library size (*rlog*) for experiments with < 30 samples. The variance stabilizing transformation (*vst)* was used to normalize transcript counts in experiments with > 30 samples [136]. IAP and apoptosis-related transcripts were subset from overall differentially expressed genes using lists of candidate genes identified above.

In order to confirm overall expression for each of the identified oyster IAP genes (i.e. to identify potential pseudogenes or genes not expressed at all in the experimental conditions included in this study), constitutive gene expression (transformed read counts) was shown for those genes containing transcripts that showed expression in all experiments but were not significantly differentially expressed in any of experiments included in the DEG analysis. Read counts for each of the genes were transformed using either the *rlog* or *vst* transformations based on sample size (the same way as above during DESeq2 analysis) and were corrected for batch effects using the limma package *‘removeBatchEffects’* [138]. Transformed read counts were averaged within each individual treatment group for each experiment.

All gene expression figures were generated in ggplot2 (V3.3.2) using “geom_tile” and compiled using cowplot (V1.0.0, Wilke, Claus). LFC heatmaps were generated with ComplexHeatmap (V 2.0.0) [139].

### Weighted gene co-expression network analysis (WGCNA)

In order to determine a potential association between IAP gene expression and expression of apoptosis-related genes, weighted gene co-expression of apoptosis genes within each individual experiment was investigated using WGCNA (V 1.68) in R (V 3.6.1) [140]. Expression data was transformed as for the DESeq2 experiment, and batch effect correction was performed the same as in the constitutive expression analysis. Network construction and module identification was performed separately for each experiment. For each network, a “signed hybrid” type network was selected and robust correlation was performed using the bi-weight mid-correlation (corFunc = “bicor”) [140]. Soft thresholding powers were set based on fit to scale free topology, or when scale free topology was not satisfied, soft thresholding was selected based on sample size (9 for “signed hybrid” with less than 30 samples). Based on results from the DEG analysis, data sets containing two genetically distinct families bred for resistance or susceptibility to disease (CVPMA, CVBAC-C, CGOSHV1-A) or distinct pathogen groups (CVBAC-A, CGBAC-A, CGBAC-B) were split for network analysis. Modules significantly correlated with immune challenge (*p*-value ≤0.05) and containing > 1 transcript for both IAP and apoptosis-related genes were analyzed. Direct correlations between apoptosis-related and IAP genes were assessed by isolating nodes where IAPs were directly connected to an apoptosis-related transcript by a shared edge. Presence and absence heatmaps for IAPs and directly correlated apoptosis-related transcripts in each experimental condition were generated with Pheatmap (V 1.0.12) [139]. Upset plots of this data were created using “UpSet” in ComplexHeatmap (V 2.0.0) and figure tables were generated using the gt package (V 0.2.1).

## Supplementary Information

**Additional file 1: Supplementary Table 1**: IAP genes and transcripts with each domain architecture manually identified in oysters (Cv = *C. virginica*, Cg = *C. gigas*). Domain architecture classification for IAPs in well supported clusters (> 90% bootstrap support) (see Fig. 4). Genes often coded for multiple IAP transcripts in each reference annotation. * = BIR Domain Identified by Interproscan and not CDD search. (XLS 28 kb)

**Additional file 2: Supplementary Table 2**: IAP gene and transcript differential expression across experiments. The number of differentially expressed transcripts, and their total number of parent genes in each experiment, added across applicable multiple comparisons made within that experiment. *Distinct refers to unique “XM” ID in that experiment. Duplicates across multiple within-experiment comparisons not counted. **Uniquely refers to those only expressed in that experiment and not expressed in any other. (XLS 29 kb)

**Additional file 3: Supplementary Table 3**: Domain architectures of significantly differentially expressed and constitutively expressed IAP transcripts in oysters. The total significantly differentially expressed and constitutively expressed transcripts per species with each domain architecture type, and the percent of the total IAPs in that species represented by that type. The total unique significantly differentially expressed transcripts with each domain architecture type in each experiment are also displayed. DEG = significantly differentially expressed genes, CEG = constitutively expressed genes. *Distinct refers to unique “XM” ID in that experiment. Duplicates across multiple within-experiment comparisons not counted. BIR* = BIR Domain Identified by Interproscan and not CDD search. (XLS 34 kb)

**Additional file 4: **S**upplementary Table 4**: Apoptosis and regulated cell death products identified in *C. gigas* and *C. virginica* reference annotations. Representative members of each apoptosis pathway-related gene product identified in *C. gigas* (Ostreida) and *C. virginica* (Ostreida) for viewing the breadth of identified products. The full list of identified apoptosis pathway related genes and transcripts for each species is presented in Additional Files 1 and 2. IAP* = Genes putatively identified as IAPs in this research based on HMMER and Interproscan analysis. (XLS 100 kb)

**Additional file 5: Supplementary Table 5**: *C. virginica* and *C. gigas* immune challenge apoptosis differential expression. The total number of differentially expressed transcripts and apoptosis-related transcripts identified in each experimental group in each experiment, and the proportion of the total differentially expressed transcripts represented by apoptosis-related transcripts. (XLS 33 kb)

**Additional file 6: Supplementary Table 6**: *C. virginica* and *C. gigas* immune challenge apoptosis co-expression. Results from Weighted Gene Correlation Network Analysis (WGCNA) for each experiment regarding: the number of significant modules, how many apoptosis-related genes and transcripts were identified across significant modules, and how many IAP genes, transcripts, and domain architectures were identified across significant modules. * = No significant modules identified by WGCNA. (XLS 29 kb)

**Additional file 7: Supplementary Table 7**: Domain architectures of IAP transcripts significantly co-expressed with apoptosis for each immune challenge. The number of IAP transcripts significantly co-expressed with each immune challenge. * = BIR Domain Identified by Interproscan and not CDD search. (XLS 29 kb)

**Additional file 8: Supplementary Table 8**: *C. virginica* IAP haplotigs identified. *C. virginica* IAP proteins identified as likely assembly artifacts (haplotigs). Read counts for IAP proteins resulting from these assembly errors were combined with their most similar protein (“Protein Haplotig Collapsed Into”) for differential expression and WGCNA analysis (see Methods). Due to lack of a chromosome-based assembly for *C. gigas* at the time this analysis was conducted, manual haplotig analysis was not performed in this species. (XLS 29 kb)

**Additional file 9: Supplementary Table 9**: *C. virginica* and *C. gigas* transcriptome experiment metadata. SRA (NCBI) database information for each transcriptome analyzed in each experiment. (XLS 50 kb)

**Additional file 10: Supplementary Table 10**: *C. virginica* and *C. gigas* transcriptome experiment DESeq2 analysis data. Metadata regarding experimental conditions for each transcriptome sample and how comparisons were designed during DESeq2 analysis to measure differential gene expression. (XLS 78 kb)

**Additional file 11: Supplementary Figure 1**: *C. virginica* IAP genomic distribution reveals potential expansion by tandem duplication and retroposition. To assess whether tandem duplication and retroposition may have contributed to *C. virginica* (Ostreida) IAP gene expansion, chromosomal locations of IAP genes, including those lacking introns, in the *C. virginica* genome were plotted as an ideogram. IAP genes are concentrated on chromosomes 6 and 7 and are present in multiple tandem arrays, suggesting tandem duplication as a mechanism of IAP expansion. Intronless IAP genes are labelled with * and are distributed on chromosomes 5, 7, 8, and 10. The presence of intronless genes suggests retroposition as a potential mechanism of IAP gene expansion in *C. virginica*. Track 1 = Chromosome length, 2 = Gene density per 1 Mb, and 3 = IAP gene location.

**Additional file 12: Supplementary Figure 2**: Classification of conserved and novel BIR-repeat domains across model organisms, *C. virginica*, *C. gigas*, and *M. yessoensis*. The sequence diversity and relationships between representative BIR domains of conserved Type I and Type II in model organisms *D. melanogaster*, *Homo sapiens*, *Mus musculus, Danio rerio*, and all BIR domain amino acid sequences identified in *C. virginica* (Ostreida), *C. gigas* (Ostreida), and *M. yessoensis* (Pectinida) were assessed by performing a phylogenetic analysis. Plotting with amino acid sequences reveals sequence diversity at conserved positions and relationships between the BIR types. (A) BIR domain-defining amino acid sequences were clustered with RAxML following MAFFT multiple sequence alignment. Sequences are labeled with their protein NCBI Accession, the sequential order of that BIR in the parent protein (i.e. BIR2 = second BIR domain from the N-terminus) and the parent gene locus. Node shapes indicate bootstrap support (circle = 90–100, upward triangle = 70–89, downward triangle = 50–69). (B) BIR domains classification was based on conservation at critical conserved residues, and BIR nodes are colored by their BIR classification type. Genus and species names for mollusc and model organism species aligned; (C) BIR domain amino acid sequences from the multiple sequence alignment plotted in the order of the RAxML tree, visualized in R using ggmsa, with critical amino acid positions highlighted in color based on their amino acid properties.

**Additional file 13: Supplementary Figure 3**: Patterns of BIR Type occurrence and loss and gain across bivalve IAPs. The sequence relationships between full IAP gene sequences from *C. virginica* (Ostreida), *C. gigas* (Ostreida) and *M. yessoensis* (Pectinida) were analyzed using a phylogenetic analysis, colored by the number of BIR domains present, and labeled with any novel BIR domains to determine any patterns of BIR domain loss and gain potentially present in the gene family across species, and whether novel BIR sequences may have arisen once or multiple times. (A) Phylogenetic tree of IAP gene sequences colored by the number of BIR domains as identified by CDD. TY = Type Y, TX = Type X, NZBIR = Non-Zinc Binding, * = Intronless. Node shapes indicate bootstrap support (circle = 90–100, upward triangle = 70–89, downward triangle = 50–69). IAP gene sequence clustering suggests a pattern of domain loss over time and independent gain of novel BIRs. (B) Number of genes in *C. gigas* and *C. virginica* with one, two, or three BIR repeats. ^1^Only genes with BIR domains confirmed by CDD were analyzed. Proteins with one and two BIR repeats were most common in studied oysters.

**Additional file 14: Supplementary Figure 4**: IAP genes and domain architectures constitutively expressed in *C. virginica* and *C. gigas*. Constitutive expression of IAP genes in each experiment was analyzed to determine whether an additional portion of IAP transcriptional diversity was important in normal physiological process not involved in immune response. This analysis revealed most domain architecture types are constitutively expressed in oysters, and thus may be important in constitutive physiological processes, rather than active disease response. (A) Phylogenetic tree of IAP amino acid sequences labelled by their gene name in *C. gigas* (Ostreida) (green), *C. virginica* (Ostreida) (blue), and *M. yessoensis* (Pectinida) (orange). A square node tip indicates collapsed *M. yessoensis* proteins for the purpose of plotting. Node shapes indicate bootstrap support (circle = 90–100, upward triangle = 70–89, downward triangle = 50–69). Vertical bars indicate well-supported protein clusters previously designated in Fig. 4. Transcripts with the same amino acid sequence were collapsed by RAxML when producing the tree. Multiple Proteins from the same gene are named once on the lowest node and then represented by dashes (“----”). (B) Heatmap of rlog or vst transformed read counts, averaged across individual treatment groups, of constitutively expressed *C. virginica* IAPs in each experiment plotted for each transcript parallel to its corresponding position on the phylogenetic tree. Shaded boxes surround each well supported protein cluster. (C) Heatmap of rlog or vst transformed read counts, averaged across individual treatment groups, of constitutively expressed *C. gigas* IAPs in each experiment plotted for each transcript parallel to its corresponding position on the phylogenetic tree. Shaded boxes surround each well supported protein cluster.

**Additional file 15: Supplementary Figure 5**: Clustering of directly correlated IAP and apoptosis-related transcripts in oysters by experiment. Direct correlations between IAP domain architecture types and apoptosis pathways identified during WGCNA were assessed for individual experiments and compared between experiments using heat map clustering to identify patterns of specific domain architectures that may be associated with specific apoptosis pathways. Results are presented as a heat map showing presence (red) or absence (blue) of correlation between IAP transcripts (y-axis; named for its domain architecture and apoptosis-related transcripts (x-axis), and the experiment in which the correlation was determined. Rather than showing any clustering between specific domain architectures and pathways, transcripts clustered mostly by experiment and not by domain architecture type.

**Additional file 16: Additional File 1**: *C. virginica* apoptosis genes, transcripts, and proteins. Text file containing GFF3 information about all identified apoptosis transcripts, genes, and proteins in the *C. virginica* reference genome annotation.

**Additional file 17: Additional File 2**: *C. gigas* apoptosis genes, transcripts, and proteins. Text file containing GFF3 information about all identified apoptosis transcripts, genes, and proteins in the *C. gigas* reference genome annotation.

**Additional file 18: Additional File 3**: Mollusc IAP Protein Multiple Sequence Alignment. FASTA file containing multiple sequence alignment of the full IAP amino acid sequences from all studied molluscs produced by MAFFT.

**Additional file 19: Additional File 4**: BIR domain Multiple Sequence Alignment. FASTA file containing Multiple Sequence alignment by MAFFT of individual BIR amino acid sequences from each protein. Sequences are named by their protein accession (XP), followed by which BIR domain the sequence was from (reading from 5′ to 3′), then ending with the gene accession (LOC). Species names for each are given in Supplementary Fig. 1b.

## Data Availability

The datasets analyzed during the current study are publicly available on the NCBI SRA (Supplementary Table 9). Mollusc reference annotations utilized are publicly available on NCBI (Table 1). Code used to perform analysis and create figures in this publication are publicly available on github (differential expression analysis and WGCNA at https://github.com/erinroberts/apoptosis_data_pipeline; apoptosis pathway and IAP annotation at https://github.com/erinroberts/Apoptosis-Pathway-Annotation-Comparative-Genomics).

## Abbreviations

A1: bcl-2-related protein A1
A20: Tumor necrosis factor alpha-induced protein 3
AIF/AIFM1: Apoptosis-inducing factor 1, mitochondrial
ATF-4: cyclic AMP-dependent transcription factor ATF-4
AURKA: Aurora kinase A
BAD: BCL2 associated agonist of cell death
BAG: BAG family molecular chaperone regulator
BAK1: bcl-2 homologous antagonist/killer
BID: BH3-interacting domain death agonist
BIK: Bcl-2-interacting killer
BIM: Bcl-2-like protein 11
BIR: Baculovirus IAP Repeat
BMF: Bcl-2-modifiying factor
BRUCE: BIR repeat-containing ubiquitin-conjugating enzyme
CARD: Caspase activation and recruitment domain
CCAR: Cell division cycle and apoptosis regulator protein 1
CD151: CD151 antigen
CDD: Conserved domain database
CDIP1: Cell death-inducing p53-target protein 1
cdc42: cdc42 homolog
CFU: Colony forming units
CHIP: E3 ubiquitin-protein ligase CHIP
CHOP: DNA damage-inducible transcript 3 protein
CRADD: Death domain-containing protein CRADD
CREB3L: Cyclic AMP-responsive element-binding protein 3-like protein
DD: Death domains
DEG: Differentially expressed genes
DEBCL: Proapoptotic Bcl-2 homolog DEBCL
DIAP1: Death-associated inhibitor of apoptosis 1
DIAP2: Death-associated inhibitor of apoptosis 2
DIABLO: Diablo homolog, mitochondrial
EIF2K3: Eukaryotic translation initiation factor 2-alpha kinase 3
DISC: Death-inducing signaling complex
DR: Death receptor
ER: Endoplasmic reticulum
FADD: Fas-associated death domain protein
FAP1: Tyrosine-protein phosphatase non-receptor type 13
FDR: False discovery rate
FLIP: FLICE inhibitory protein
FREP: Fibrinogen-related protein
FSW: Filtered sterile seawater
GADD: Growth arrest and DNA damage-inducible protein GADD
GIMAP: GTPase IMAP family member
HK1: Hexokinase-1
HRK: Activator of apoptosis harakiri
IAP: Inhibitor of apoptosis protein
IFI27: Interferon alpha-inducible protein 27, mitochondrial
IFI44: Interferon-induced protein 44
IFN: Interferon
IL17: Interleukin 17-like protein
IL17RD: Interleukin-17 receptor D
IRAK4: Interleukin-1 receptor-associated kinase 4
IRE1: serine/threonine-protein kinase/endoribonuclease IRE1
IRF: Interferon regulatory factor 1
IRF8: Interferon regulatory factor 8
JAK2: Tyrosine-protein kinase JAK2
LFC: log fold change
LITAF: Lipopolysaccharide-induced tumor necrosis factor-alpha factor
LPS: Lipopolysaccharide-induced tumor necrosis factor-alpha factor
LTR: Long terminal repeat
MAPK: Mitogen-activated protein kinase
MIF: Macrophage migration inhibitory factor
MyD88: Myeloid differentiation primary response protein MyD88
NOXA: phorbol-12-myristate-13-acetate- induced protein 1
NR13: anti-apoptotic protein NR13
ORF: Open reading frame
PARP1: Poly [ADP-ribose] polymerase 1
PDRG1: p53 and DNA damage-regulated protein 1
PIDD1: leucine-rich repeat and death domain-containing protein 1
PPM1B: Protein phosphatase 1B
PRR: pattern recognition receptor
RCD: Regulated cell death
RHOT1: mitochondrial Rho GTPase 1
RING: really interesting new gene
RIPK: receptor-interacting serine/threonine-protein kinase
SARM1: sterile alpha and TIR motif-containing protein 1
SERPINB1: leukocyte elastase inhibitor
STAT: Signal transducer and activator of transcription 5A
STING: Stimulator of interferon genes protein
TAB1: TGF-beta-activated kinase 1 and MAP3K7-binding protein 1
TAK1: mitogen-activated protein kinase kinase kinase 7
TLR: Toll-like receptor
TNF: Tumor necrosis factor
TNFAIP: Tumor necrosis factor alpha-induced protein
TNFR: Tumor necrosis factor receptor
TNFRSF: Tumor necrosis factor receptor superfamily member
TRADD: Tumor necrosis factor receptor superfamily member 5
TRAF: TNF receptor-associated factor
TRAIL: tumor necrosis factor ligand superfamily member 10
UBA: Ubiquitin associated domain
UBC: Ubiquitin conjugating domain
XIAP: E3 ubiquitin-protein ligase XIAP
