# Supplementary figures and images for: The expanded inhibitor of apoptosis gene family in oysters possesses novel domain architectures and may play diverse roles in apoptosis following immune challenge
Source: BMC Genomics. 2022 Mar 12;23:201. doi: 10.1186/s12864-021-08233-6 (PMC8917759; doi:10.1186/s12864-021-08233-6)

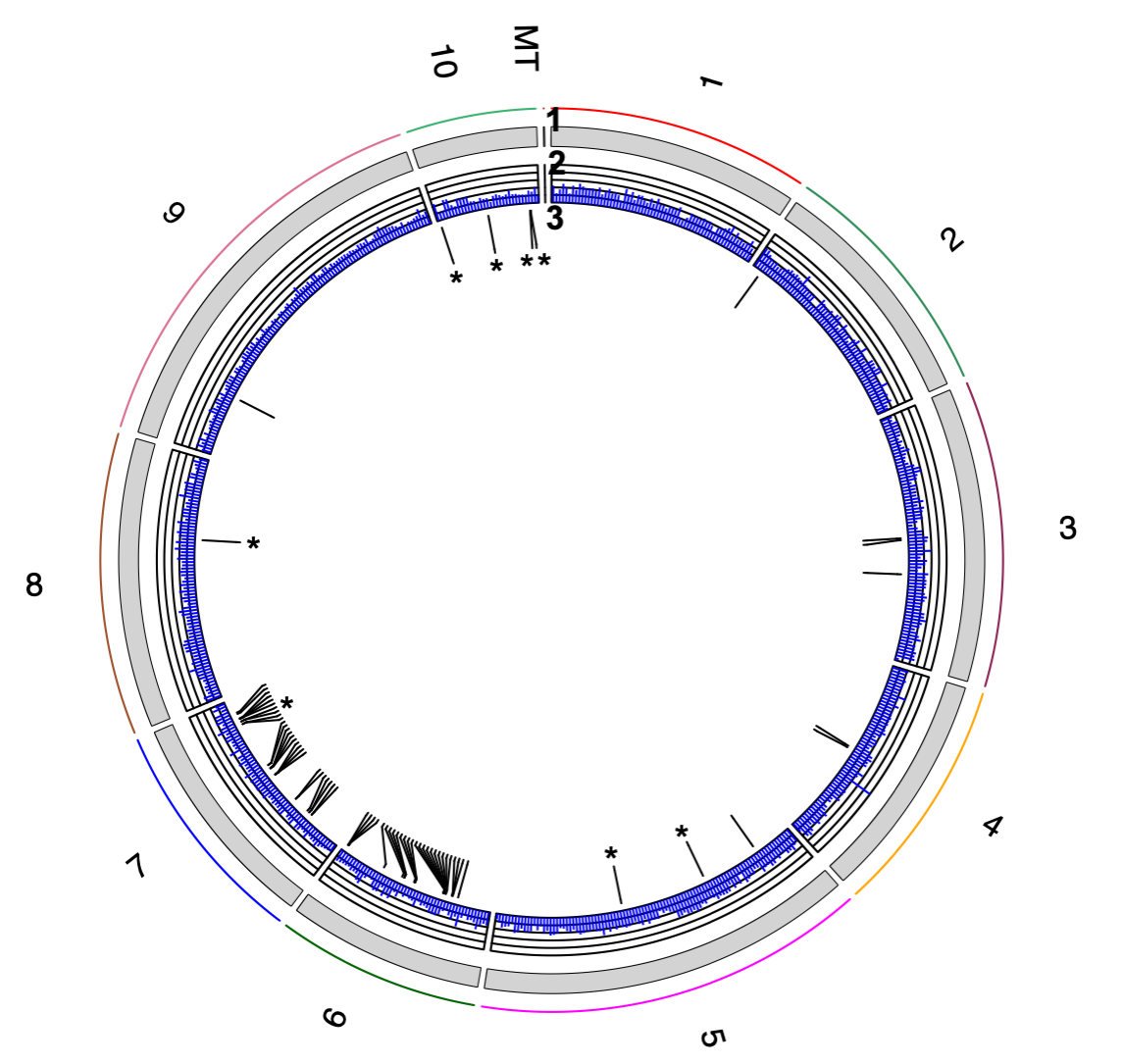

Supplement: Supplementary file 11 — Additional file 11: Supplementary Figure 1: C. virginica IAP genomic distribution reveals potential expansion by tandem duplication and retroposition. To assess whether tandem duplication and retroposition may have contributed to C. virginica (Ostreida) IAP gene expansion, chromosomal locations of IAP genes, including those lacking introns, in the C. virginica genome were plotted as an ideogram. IAP genes are concentrated on chromosomes 6 and 7 and are present in multiple tandem arrays, suggesting tandem duplication as a mechanism of IAP expansion. Intronless IAP genes are labelled with * and are distributed on chromosomes 5, 7, 8, and 10. The presence of intronless genes suggests retroposition as a potential mechanism of IAP gene expansion in C. virginica. Track 1 = Chromosome length, 2 = Gene density per 1 Mb, and 3 = IAP gene location. [file 12864_2021_8233_MOESM11_ESM.png]
