# Supplementary material for: The expanded inhibitor of apoptosis gene family in oysters possesses novel domain architectures and may play diverse roles in apoptosis following immune challenge
Source: BMC Genomics. 2022 Mar 12;23:201. doi: 10.1186/s12864-021-08233-6 (PMC8917759; doi:10.1186/s12864-021-08233-6)
Supplement: Supplementary file 13 — Additional file 13: Supplementary Figure 3: Patterns of BIR Type occurrence and loss and gain across bivalve IAPs. The sequence relationships between full IAP gene sequences from C. virginica (Ostreida), C. gigas (Ostreida) and M. yessoensis (Pectinida) were analyzed using a phylogenetic analysis, colored by the number of BIR domains present, and labeled with any novel BIR domains to determine any patterns of BIR domain loss and gain potentially present in the gene family across species, and whether novel BIR sequences may have arisen once or multiple times. (A) Phylogenetic tree of IAP gene sequences colored by the number of BIR domains as identified by CDD. TY = Type Y, TX = Type X, NZBIR = Non-Zinc Binding, * = Intronless. Node shapes indicate bootstrap support (circle = 90–100, upward triangle = 70–89, downward triangle = 50–69). IAP gene sequence clustering suggests a pattern of domain loss over time and independent gain of novel BIRs. (B) Number of genes in C. gigas and C. virginica with one, two, or three BIR repeats. 1Only genes with BIR domains confirmed by CDD were analyzed. Proteins with one and two BIR repeats were most common in studied oysters. [file 12864_2021_8233_MOESM13_ESM.pdf]

A

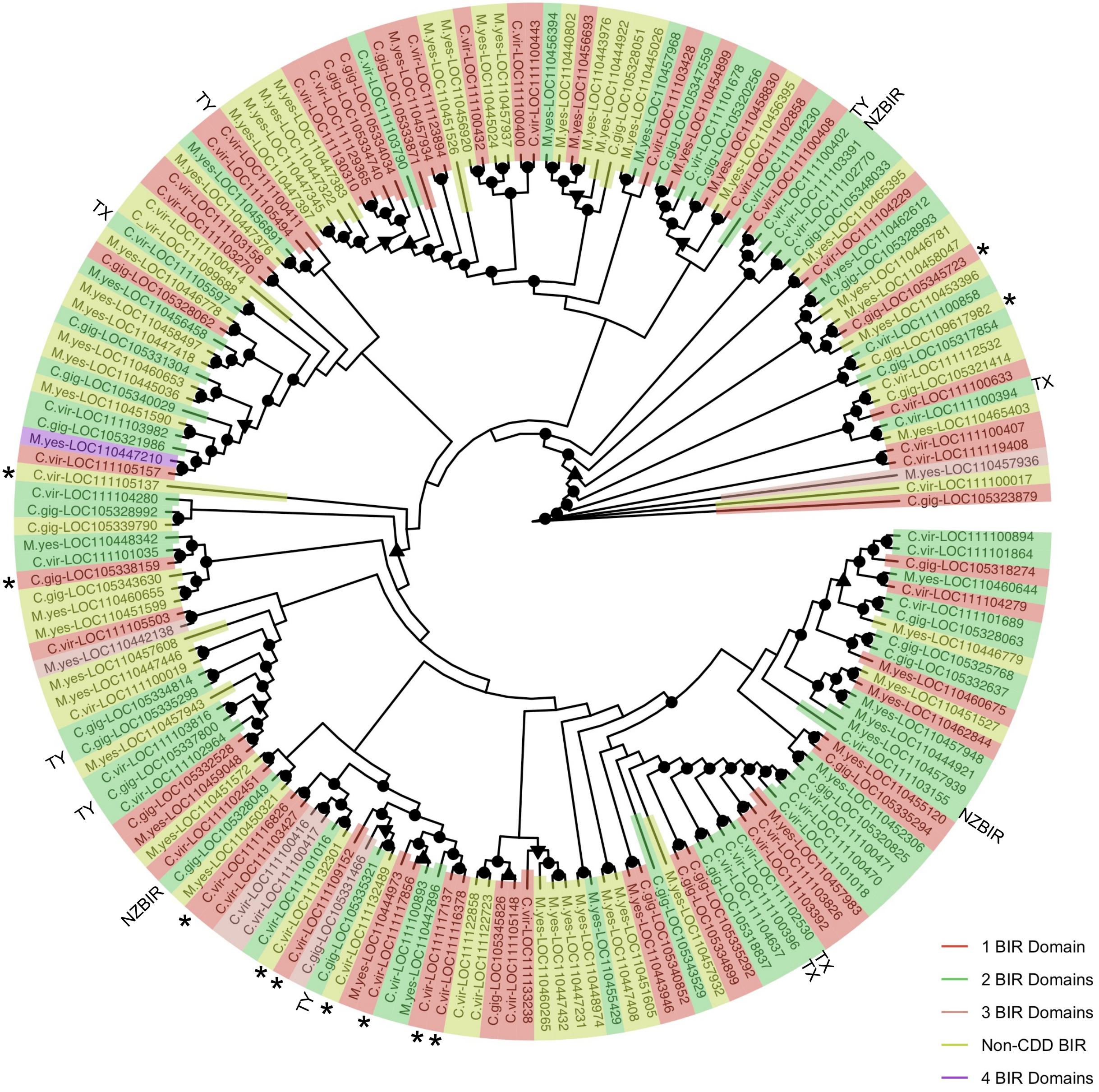

B

BIR Number	Number of Genes *C. vir*[1]	*C. gig*[1]	*M. yes*[1]
N — BIR — C	*31*	*14*	*11*
N — BIR — BIR — C	*26*	*20*	*13*
N — BIR — BIR — BIR — C	*2*	*1*	*2*
