# Supplementary material for: The expanded inhibitor of apoptosis gene family in oysters possesses novel domain architectures and may play diverse roles in apoptosis following immune challenge
Source: BMC Genomics. 2022 Mar 12;23:201. doi: 10.1186/s12864-021-08233-6 (PMC8917759; doi:10.1186/s12864-021-08233-6)
Supplement: Supplementary file 15 — Additional file 15: Supplementary Figure 5: Clustering of directly correlated IAP and apoptosis-related transcripts in oysters by experiment. Direct correlations between IAP domain architecture types and apoptosis pathways identified during WGCNA were assessed for individual experiments and compared between experiments using heat map clustering to identify patterns of specific domain architectures that may be associated with specific apoptosis pathways. Results are presented as a heat map showing presence (red) or absence (blue) of correlation between IAP transcripts (y-axis; named for its domain architecture and apoptosis-related transcripts (x-axis), and the experiment in which the correlation was determined. Rather than showing any clustering between specific domain architectures and pathways, transcripts clustered mostly by experiment and not by domain architecture type. [file 12864_2021_8233_MOESM15_ESM.pdf]

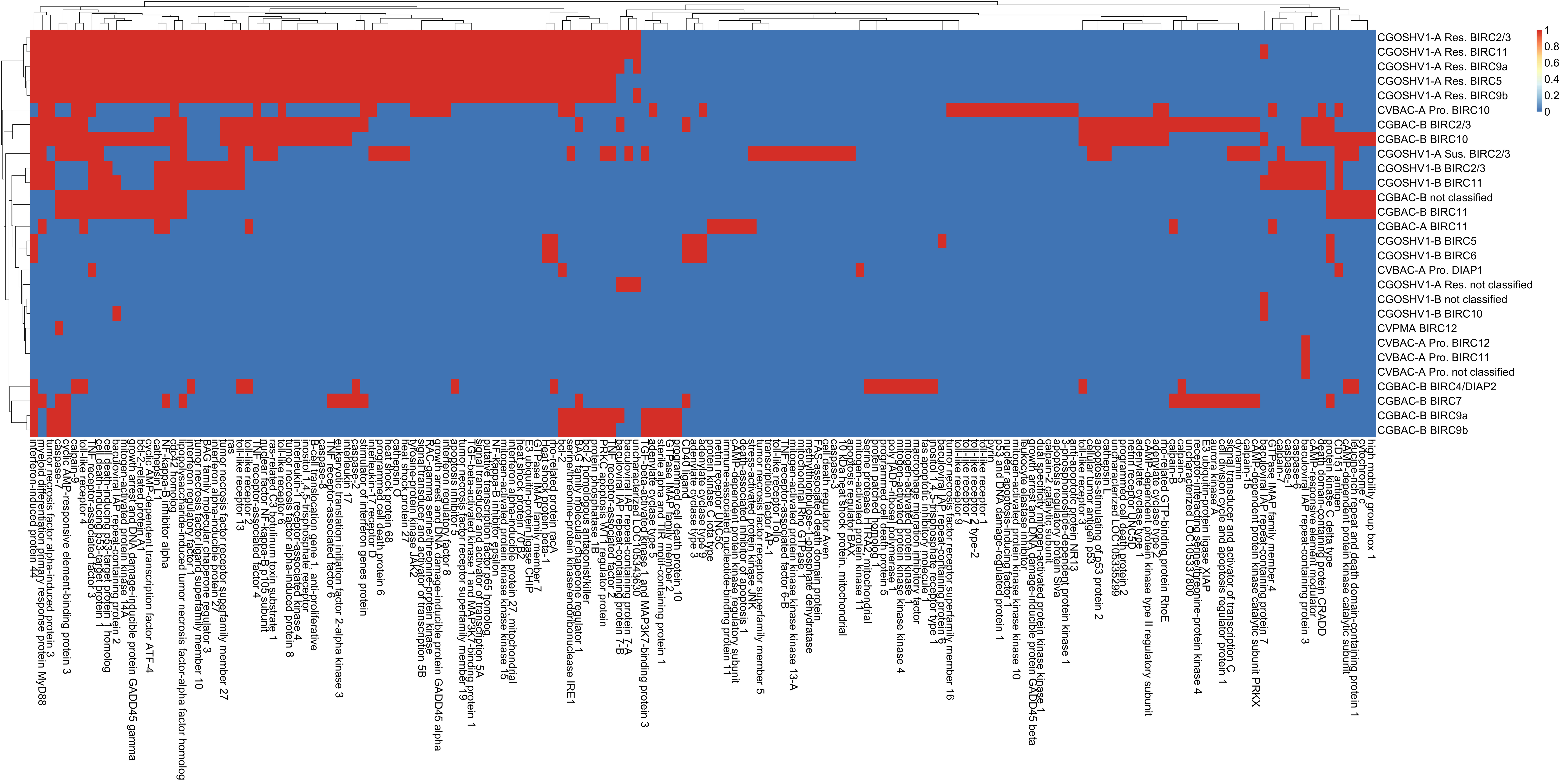

1
0.8
0.6
0.4
0.2
0
CGOSHV1-A Res. BIRC2/3
CGOSHV1-A Res. BIRC11
CGOSHV1-A Res. BIRC9a
CGOSHV1-A Res. BIRC5
CGOSHV1-A Res. BIRC9b
CVBAC-A Pro. BIRC10
CGBAC-B BIRC2/3
CGBAC-B BIRC10
CGOSHV1-A Sus. BIRC2/3
CGOSHV1-B BIRC2/3
CGOSHV1-B BIRC11
CGBAC-B not classified
CGBAC-B BIRC11
CGBAC-A BIRC11
CGOSHV1-B BIRC5
CGOSHV1-B BIRC6
CVBAC-A Pro. DIAP1
CGOSHV1-A Res. not classified
CGOSHV1-B not classified
CGOSHV1-B BIRC10
CVPMA BIRC12
CVBAC-A Pro. BIRC12
CVBAC-A Pro. BIRC11
CVBAC-A Pro. not classified
CGBAC-B BIRC4/DIAP2
CGBAC-B BIRC7
CGBAC-B BIRC9a
CGBAC-B BIRC9b
interferon-induced protein 44
myeloid differentiation primary response protein MyD88
tumor necrosis factor alpha-induced protein 3
caspase-7
cyclic AMP-responsive element-binding protein 3
calpain-9
toll-like receptor 4
TNF receptor-associated factor 3
cell death-inducing p53-target protein 1
cell death-inducing p53-target protein 1 homolog
baculoviral IAP repeat-containing protein 2
mitogen-activated protein kinase 14A
growth arrest and DNA damage-inducible protein GADD45 gamma
bcl-2-related protein A1
cyclic AMP-dependent transcription factor ATF-4
cathepsin L1
NF-kappa-B inhibitor alpha
cdc42 homolog
lipopolysaccharide-induced tumor necrosis factor-alpha factor homolog
interferon regulatory factor 1
interferon alpha-inducible protein 27
BAG family molecular chaperone regulator 3
tumor necrosis factor ligand superfamily member 10
tumor necrosis factor receptor superfamily member 27
ras
toll-like receptor 13
toll-like receptor 6
TNF receptor-associated factor 4
nuclear factor NF-kappa-B p105 subunit
ras-related C3 botulinum toxin substrate 1
tumor necrosis factor alpha-induced protein 8
interleukin-1 receptor-associated kinase 4
inositol 1,4,5-trisphosphate receptor
B-cell translocation gene 1, anti-proliferative
caspase-8
TNF receptor-associated factor 6
eukaryotic translation initiation factor 2-alpha kinase 3
interleukin 17
caspase-2
interleukin-17 receptor D
stimulator of interferon genes protein
programmed cell death protein 6
heat shock protein 68
cathepsin O
heat shock protein 27
tyrosine-protein kinase JAK2
signal transducer and activator of transcription 5B
RAC-gamma serine/threonine-protein kinase
growth arrest and DNA damage-inducible protein GADD45 alpha
interferon regulatory factor 8
apoptosis inhibitor 5
tumor necrosis factor receptor superfamily member 19
TGF-beta-activated kinase 1 and MAP3K7-binding protein 1
signal transducer and activator of transcription 5A
putative transcription factor p65 homolog
NF-kappa-B inhibitor epsilon
mitogen-activated protein kinase kinase kinase 15
interferon alpha-inducible protein 27, mitochondrial
heat shock protein 70B2
E3 ubiquitin-protein ligase CHIP
GTPase IMAP family member 7
heat shock protein beta-1
rho-related protein racA
bcl-2
serine/threonine-protein kinase/endoribonuclease IRE1
BAG family molecular chaperone regulator 1
bcl-2 homologous antagonist/killer
protein phosphatase 1B
PRKC apoptosis WT1 regulator protein
TNF receptor-associated factor 2
baculoviral IAP repeat-containing protein 7-A
baculoviral IAP repeat-containing protein 7-B
uncharacterized LOC105343630
TGF-beta-activated kinase 1 and MAP3K7-binding protein 3
adenylate cyclase type 5
sterile alpha and TIR motif-containing protein 1
GTPase IMAP family member 2
programmed cell death protein 10
CD40 ligand
adenylate cyclase type 3
adenylate cyclase type 9
protein kinase C iota type
netrin receptor UNC5C
cAMP-dependent protein kinase regulatory subunit
immune-associated nucleotide-binding protein 11
death-associated inhibitor of apoptosis 1
stress-activated protein kinase JNK
tumor necrosis factor receptor superfamily member 5
transcription factor AP-1
toll-like receptor Tollo
TNF receptor-associated factor 6-B
mitogen-activated protein kinase kinase kinase 13-A
mitochondrial Rho GTPase 1
methylthioribulose-1-phosphate dehydratase
FAS-associated death domain protein
cell death regulator Aven
caspase-3
apoptosis regulator BAX
10 kDa heat shock protein, mitochondrial
mitogen-activated protein kinase 11
serine protease HTRA2, mitochondrial
protein patched homolog 1
programmed cell death protein 5
poly [ADP-ribose] polymerase 1
mitogen-activated protein kinase 1
mitogen-activated protein kinase kinase 4
macrophage migration inhibitory factor
fas apoptotic inhibitory molecule 1
inositol 1,4,5-trisphosphate receptor type 1
baculoviral IAP repeat-containing protein 6
tumor necrosis factor receptor superfamily member 16
toll-like receptor 9
toll-like receptor 7
toll-like receptor 2 type-2
toll-like receptor 1
pyrin
p53 and DNA damage-regulated protein 1
nuclear apoptosis-inducing factor 1
mitogen-activated protein kinase kinase 10
leukocyte elastase inhibitor
growth arrest and DNA damage-inducible protein GADD45 beta
dual specificity mitogen-activated protein kinase kinase 1
calpain-2 catalytic subunit
apoptosis regulatory protein Siva
3-phosphoinositide-dependent protein kinase 1
anti-apoptotic protein NR13
toll-like receptor 3
cellular tumor antigen p53
cathepsin L
apoptosis-stimulating of p53 protein 2
uncharacterized LOC105335299
programmed cell death protein 2
netrin receptor UNC5D
adenylate cyclase type 1
cAMP-dependent protein kinase type II regulatory subunit
adenylate cyclase type 2
rho-related GTP-binding protein RhoE
calpain-B
calpain-5
uncharacterized LOC105337800
receptor-interacting serine/threonine-protein kinase 4
E3 ubiquitin-protein ligase XIAP
aurora kinase A
cell division cycle and apoptosis regulator protein 1
signal transducer and activator of transcription C
dynamin
calpain-15
cAMP-dependent protein kinase catalytic subunit PRKX
baculoviral IAP repeat-containing protein 7
GTPase IMAP family member 4
calpain-7
caspase-1
caspase-6
baculoviral IAP repeat-containing protein 3
cAMP-responsive element modulator
death domain-containing protein CRADD
protein kinase C delta type
CD151 antigen
cAMP-dependent protein kinase catalytic subunit
leucine-rich repeat and death domain-containing protein 1
cytochrome c
high mobility group box 1
